# The effects of taekwondo on depression symptoms and cognitive function: a systematic review and meta-analysis

**DOI:** 10.3389/fspor.2025.1735531

**Published:** 2026-01-15

**Authors:** Aijiao Chen, Xin Tian, Xiujie Ma

**Affiliations:** 1 School of Wushu, Chengdu Sport University, Chengdu, China; 2 Organization Department, Chengdu Sport University, Chengdu, China; 3 Chinese GuoShu Academy, Chengdu Sports University, Chengdu, Sichuan, China

**Keywords:** cognitive function, depression, meta-analysis, systematic review, taekwondo

## Abstract

**Background:**

Depression is among the most prevalent mental disorders globally and is frequently accompanied by impairments in attention, memory, and executive functioning that substantially diminish quality of life and social functioning. Taekwondo has increasingly been recognized as a holistic mind–body discipline that integrates physical training, attentional regulation, and ethical cultivation and may offer benefits for both psychological well-being and cognitive health. However, the existing evidence has not been systematically synthesized.

**Methods:**

This systematic review and meta-analysis was conducted in accordance with PRISMA guidelines and synthesized relevant literature published through September 2025. Searches were conducted across major English- and Chinese-language Taekwondodatabases (PubMed, Embase, Cochrane Library, Web of Science, Ovid Medline and CNKI), and 14 randomized controlled trials were ultimately included. Two reviewers independently screened studies, extracted data, and assessed risk of bias using the RoB 2.0 tool, and rated the certainty of evidence using the GRADE approach. Random-effects models were applied to estimate standardized mean difference (SMD) and corresponding 95% CI. Heterogeneity was examined using Cochran's *Q*-test and the *I*^2^ statistic. Subgroup analyses and meta-regression were performed to evaluate the moderating effects of intervention duration, weekly frequency, session length, and gender ratio. Publication bias was assessed using Begg and Egger tests, and sensitivity analyses were conducted.

**Results:**

Fourteen randomized controlled trials involving 906 participants were included. Taekwondo training was associated with significant, moderate improvements in depressive symptoms (SMD = −0.54; 95% CI: −0.84 to −0.24; *I*^2^ = 59.2%) and cognitive function (SMD = 0.49; 95% CI: 0.17–0.81; *I*^2^ = 51.4%). Subgroup analyses indicated that interventions delivered ≥3 times per week, with total durations ≤12 weeks and session lengths ≤40 min, produced the greatest improvements in depressive symptoms. In contrast, interventions delivered ≥3 times per week, with sessions lasting 30–50 min and durations exceeding 16 weeks, were more effective in enhancing cognitive function. More consistent treatment effects were observed among female participants.

**Conclusion:**

Taekwondo training appears to exert meaningful benefits for both depressive symptoms and cognitive function, supporting its potential as a comprehensive exercise-based intervention with psychological and cognitive health value.

## 1 Introduction

Depression is among the most prevalent mental disorders worldwide and remains a leading cause of disability. According to data from the World Health Organization, approximately 332 million people are expected to experience depression by 2025 (1). The core characteristics of depression include a persistent low mood and anhedonia (2). Of greater concern, depression frequently impairs cognitive function (3), resulting in broad declines in attention, working memory, executive function, and information-processing speed (4, 5), which substantially diminish social functioning and quality of life. Existing research indicates that individuals with major depressive disorder exhibit cognitive impairments in these domains (6). Crucially, such deficits often persist beyond the acute episode; substantial evidence shows that even after partial or complete remission, many individuals continue to exhibit varying degrees of “cognitive residual symptoms,” which impede the recovery of daily functioning, reintegration into social roles, and effective management of relapse risk (7, 8). Consequently, treatments that target affective symptoms alone are often insufficient for achieving comprehensive recovery. Moreover, standard pharmacological and psychotherapeutic interventions produce limited and variable effects on both depressive symptoms and cognitive functioning, underscoring the need to explore multimodal intervention approaches.

Physical activity is widely recognized as a potent non-pharmacological intervention for improving mental health and cognitive function (9, 10), and recent frameworks have further highlighted its role as an integral component in innovative strategies for suicide prevention and depression management (11). Among various forms of exercise, Taekwondo—an integrated martial art combining physical training with traditional cultural elements—has been increasingly regarded as a promising holistic intervention. Compared with other physical activities, Taekwondo entails more complex movement patterns and reaction tasks, emphasizing the cultivation of willpower and behavioral discipline through concentration, meditation, and breath control (12). These practices promote self-control and emotional regulation, thereby alleviating negative emotions such as depression and anxiety (13). Empirical evidence has confirmed that participation in Taekwondo improves mental health across diverse populations (14, 15). Long-term and regular Taekwondo training not only reduces depressive and anxiety symptoms but also strengthens self-efficacy and emotional regulation (16). Recent studies have demonstrated the benefits of Taekwondo training on executive function and cognitive control abilities in participants (17, 18). These findings are supported by exercise-induced brain plasticity theory, which posits that long-term, complex physical activities systematically trigger adaptive remodeling of brain structure and function (19). At the behavioral level, extensive research has confirmed that Taekwondo effectively enhances executive function, working memory, and cognitive control across diverse participant groups, including healthy males and middle-aged/elderly females (20, 21).

Numerous randomized controlled trials have demonstrated the positive effects of Taekwondo interventions on cognitive function and emotional well-being. However, academic consensus regarding Taekwondo's impact on cognitive abilities remains elusive. Research indicates that regular Taekwondo training can improve mental health and enhance cognitive function (22, 23). Recent studies, however, challenge this view, suggesting that while Taekwondo training may moderately improve emotional regulation, its impact on cognitive function is not significant (24, 25). These divergent findings likely stem from substantial heterogeneity in existing studies regarding intervention dosage (e.g., training intensity, frequency, duration), study populations (e.g., age, health status), and measured cognitive dimensions. These sources of heterogeneity have not been systematically addressed in current reviews. Therefore, a deeper systematic analysis is needed to more specifically and comprehensively evaluate the effects of Taekwondo training on depression and cognitive function, ensuring more accurate and reliable conclusions.

Although preliminary research has demonstrated the positive effects of Taekwondo on depression and cognitive function, existing systematic reviews and meta-analyses still exhibit several limitations. Therefore, this study employs systematic review and meta-analysis methods to comprehensively analyze all randomized controlled trials examining the effects of Taekwondo on depression and cognitive function. This aims to provide theoretical support and practical guidance on the overall efficacy of Taekwondo intervention in addressing depression and cognitive health.

## 2 Materials and methods

This systematic review and meta-analysis was conducted in accordance with established methodological guidance for evidence synthesis, primarily the Cochrane Handbook for Systematic Reviews of Interventions. The reporting of the review adhered to the Preferred Reporting Items for Systematic Reviews and Meta-Analyses (PRISMA) 2020 statement to ensure transparency and completeness. In addition, we used the GRADE approach to rate the overall certainty of evidence for each primary outcome, with detailed ratings presented in Supplementary S8. This review was conducted in compliance with the Preferred Reporting Items for Systematic Reviews and Meta-Analyses (PRISMA) statement (26). The complete PRISMA checklist is provided in Supplementary S1, demonstrating compliance with all relevant reporting requirements. The protocol for this systematic review has been registered with PROSPERO (CRD 420251173580). Throughout the review process, all steps outlined in the PRISMA statement were strictly adhered to, ensuring transparency.

### 2.1 Search strategy

We conducted database searches of Chinese and English databases including PubMed, Embase, Cochrane Library, Web of Science, Ovid Medline, and CNKI. The search was not restricted by language or publication date, with the final search completed on September 20, 2025. The search strategy incorporated relevant Medical Subject Headings (MeSH) and free-text terms—such as “Taekwondo”, “Martial Arts”, “Executive Function”, “Cognition”, “Mental Health”, and “Depression”—combined using Boolean operators (AND, OR). For example, the PubMed search string included: (“Taekwondo” AND “Depression” AND “Cognition”). Although no language restrictions were applied, only peer-reviewed articles were included. Further details of the search strategy are provided in Supplementary S2.

### 2.2 Inclusion and exclusion criteria

The inclusion criteria were defined according to the PICOS framework—Population, Intervention, Comparator, Outcomes, and Study Design. Population criteria encompassed studies involving any group, including children, adolescents, adults, and clinical cohorts. The intervention of interest was Taekwondo used as the primary intervention. Comparator conditions included alternative interventions or no intervention, such as waiting-list controls or standard care. Outcomes required the reporting of mental health or cognitive indicators, including depressive symptoms, attention, memory, or executive functioning. Eligible study designs were randomized controlled trials (RCTs) published in peer-reviewed journals, with publications dated on or before 20 September 2025, a restriction that was adopted to maximise internal validity but that may have reduced the number and scope of eligible studies.

Exclusion criteria included observational or cross-sectional studies, non–peer-reviewed publications (e.g., conference abstracts), review articles, studies for which the full text was unavailable, duplicate records, and studies with insufficient methodological or outcome reporting.

The two authors independently screened studies according to inclusion and exclusion criteria by reviewing titles and abstracts, removing duplicates, and discussing to determine final eligibility. In cases of disagreement during screening, a third author (Ma) was consulted to resolve the issue.

### 2.3 Data extraction

Two authors from the research team independently reviewed and extracted specific information from the identified studies. Each author extracted the following information from the included studies: (1) Basic study details (e.g., first author, publication year, country of study, sample size); (2) Participants' characteristics (e.g., age, gender); (3) Intervention details (e.g., program, duration, frequency per week, session length); (4) Tools used to assess participants' mental health and cognitive function; (5) Outcome measures. For studies reporting multiple outcomes, the most representative indicator was extracted. Specifically, for studies using the color–word interference test, accuracy was selected as the primary indicator because it best reflects cognitive control ability (27). The quality (certainty) of evidence for each pooled outcome was subsequently assessed using the GRADE (Grading of Recommendations Assessment, Development, and Evaluation) approach, which evaluates evidence based on risk of bias, inconsistency, indirectness, imprecision, and publication bias; the full GRADE evidence profile is provided in Supplementary S8. Discrepancies were discussed with a third author (Ma) to reach consensus.

### 2.4 Quality assessment

The quality of each included study was assessed by two authors. Data related to participants, interventions, and outcomes were independently extracted by two authors using pre-specified data extraction forms. Risk of bias assessments were concurrently conducted using the Cochrane Risk of Bias tool (RoB 2.0). The RoB 2.0 tool assessed five domains of potential bias: (1) bias in the randomization process; (2) bias in allocation to intervention; (3) bias from missing outcome data; (4) bias in outcome measurement; (5) bias in selection of reported outcomes. In accordance with the Cochrane Handbook for Systematic Reviews, each domain and the overall risk of bias were rated as “low risk,” “moderate risk,” or “high risk.” Disagreements between the two authors during the assessment were resolved through discussion with a third author (Ma). In a second step, we summarised the overall certainty of the body of evidence for depressive symptoms and cognitive outcomes using the GRADE framework, drawing on the risk-of-bias assessments and other domains specified in the GRADE guidance (see Supplementary S8).

### 2.5 Statistical analysis

Statistical analyses were performed following methodological recommendations outlined in the Cochrane Handbook, including the selection of random-effects models, heterogeneity assessment, and exploration of potential moderators. The Cochrane Risk of Bias tool (RoB 2.0) was employed to assess the methodological quality of the included studies. Data integration, heterogeneity testing, forest plot generation, and publication bias analysis were performed using Stata 17.0. As most mental health outcome measures in the included studies were continuous variables with varying measurement tools and scale units, the standardized mean difference (SMD) and its 95% confidence interval (95% CI) were used as the composite effect size measure. Heterogeneity was assessed using Cochran's *Q*-test and the *I*^2^ statistic. A significant *Q*-test value may indicate heterogeneity, while *I*^2^ reflects its magnitude, with values of 25%, 50%, and 75% representing low, moderate, and high heterogeneity, respectively (28, 29). When substantial heterogeneity was detected, random-effects models were applied. Publication bias was evaluated using Begg's rank correlation test and Egger's regression test, supplemented by visual inspection of funnel plots (Supplementary S3, S4). Sensitivity analyses were performed to determine whether individual studies acted as statistical outliers influencing the pooled results (Supplementary S5, S6); although several studies reported relatively large SMD, none exerted undue influence, and therefore all studies were retained in the primary analysis. To assess whether differences in effect sizes across subgroups were statistically significant, meta-regression using restricted maximum likelihood estimation was conducted within a random-effects model. Because Q-between statistics are applicable only to fixed-effects models, Wald *F*-tests with Knapp–Hartung correction were used to test subgroup differences. This approach provided a robust evaluation of whether intervention duration, weekly frequency, session length, and gender composition moderated the observed effect sizes.

## 3 Results

### 3.1 Studies selection

**Figure 1:**
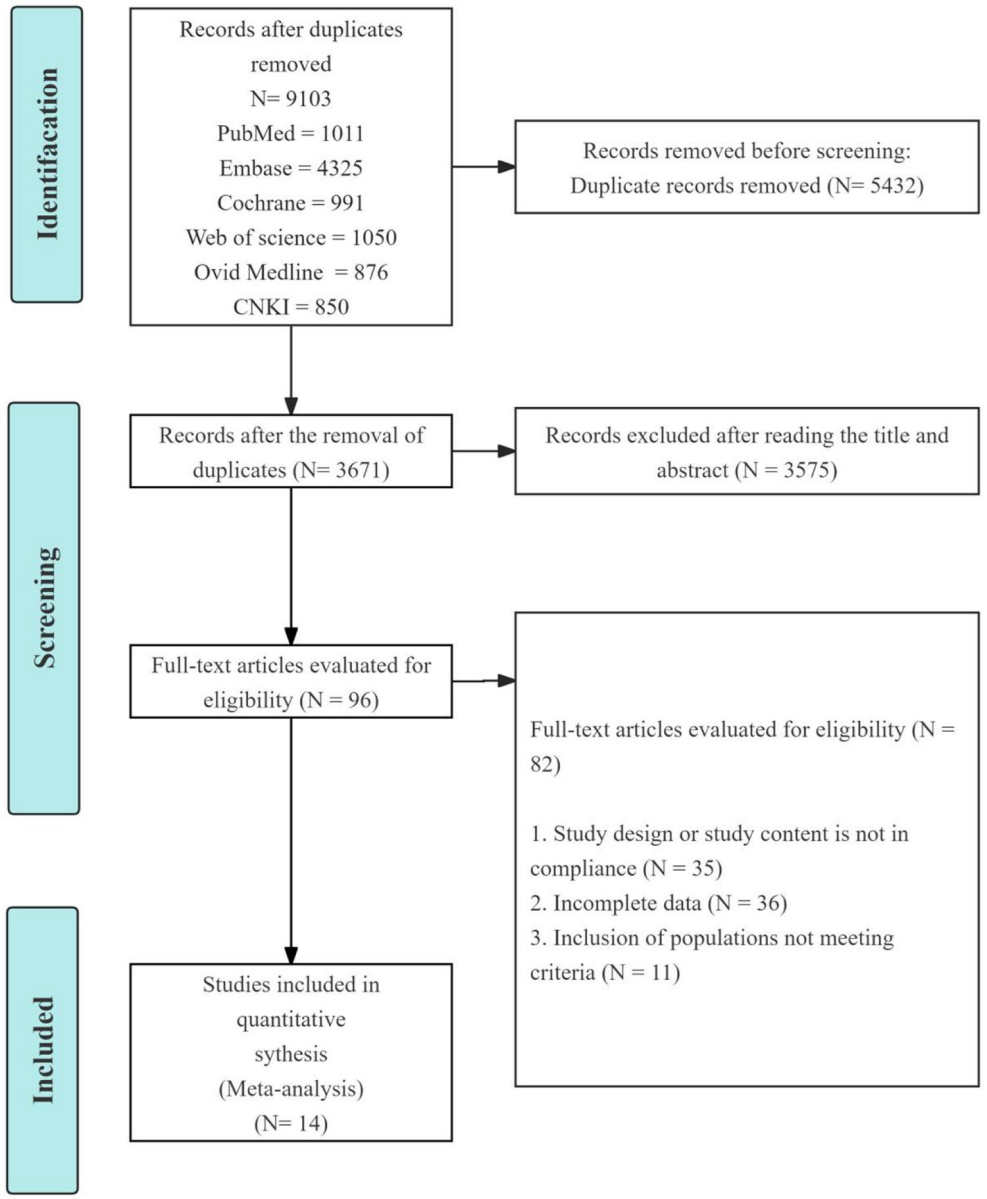
Flow of study selection.

Database searches were conducted in PubMed, Embase, Cochrane Library, Web of Science, Ovid Medline and CNKI as of September 20, 2025. A total of 9,103 publications were retrieved from these databases. During the initial screening, duplicates were removed, and titles and abstracts were reviewed, excluding 9,089 studies. Of these 5,432 studies were duplicates, and 3,575 studies were deemed irrelevant. The remaining 96 publications underwent full-text review, leading to the exclusion of 82 studies for various reasons: non-compliant research content (*n* = 35); lack of outcome measures (*n* = 36); and inclusion of populations not meeting the criteria (*n* = 11). Fourteen meta-analysis publications were also screened, as shown in Figure 1. Fourteen experiments provided data for the meta-analysis, and their characteristics are summarized in Table 1.

**Table 1:** Included trial characteristics.

Trial ID	Country	Participants	*N*	Age mean (range)	Gender, % Female	T	C	outcome measures	Research Type
Kadri et al. (30)	Tunisia	Adolescents with ADHD	40	11–17	10	20	20	SC-WT	RCT
Cho et al. (21)	Korea	Older Women	37	65–73	100	19	18	SC-WT	RCT
Roh et al. (31)	Korea	Children	30	9–12	40	15	15	K-POMS-B	RCT
								SC-WT	
Yun et al. (32)	China	College students	41	18–25	13.33	15	15	BDI-II	RCT
								RIT	
Cho et al. a (33)	Korea	Elementary school student	30	9–11	50	15	15	SC-WT	RCT
Lakes et al. (34)	America	Junior high school student	28	8–11	14.29	14	14	HFT	RCT
Bae et al. (25)	Korea	College students	24	18–23	41.67	12	12	K-POMS-B	RCT
								SC-WT	
Cho et al. b (35)	Korea	Elementary school student	35	10–12	40	17	18	SC-WT	RCT
Moon et al. (36)	Korea	Elderly	30	70–80	100	15	15	K-MMSE	RCT
Jung et al. (37)	Korea	Elderly	20	65–70	100	10	10	K-GDS	RCT
								K-MMSE	
Kim et al. (38)	Korea	Older women	23	68–76	100	11	12	K-GDS	RCT
								K-MMSE	
Kang (39)	China	Junior high school students	200	13–15	50	100	100	SCL-90	RCT
Wang (40)	China	Junior high school students	50	12–15	50	25	25	MSSMHS	RCT
Zhang et al. (41)	China	College students	255	17–19	60.78	155	100	SDS	RCT

ADHD, Attention Deficit Hyperactivity Disorder; SC – WT, Stroop Color-Word Test; HFT, Hearts & Flowers Test; K-POMS-B, Korean Profile of Mood States-Brief; BDI-II, Beck Depression Inventory-II; RIT, Response Inhibition Task; K-MMSE, Korean Mini-Mental State Examination; K-GDS, Korean Geriatric Depression Scale; SCL-90, Symptom Checklist-90; MSSMHS, Middle School Students' Mental Health Scale; SDS, Self-Rating Depression Scale; RCT, Randomized controlled trial.

### 3.2 Characteristics of eligible studies

A total of 14 publicly published papers were included in our systematic review. Of these, two papers were published in Korean (37, 38), three in Chinese, and the remaining nine in English. The studies were conducted in China (32, 39–41), Korea (21, 25, 31, 33, 35–38), America (34), and Tunisia (30). The number of participants varied across studies, ranging from 20 to 255, with a total of 906 participants aged between 5 and 80 years. All included studies employed randomized controlled trial designs. Four studies assessed only depression outcomes, six assessed only cognitive function outcomes, and the remaining studies examined both depression and cognitive function simultaneously.

### 3.3 Interventions and controls

Table 2 presents the characteristics of Taekwondo interventions across all included studies. The intervention group consistently engaged in Taekwondo training. For the control groups, eight studies used conventional physical education classes, while others employed waiting-list controls (WLC), no-training groups, or educational program groups. Interventions varied in both duration and intensity. The total intervention periods ranged from a minimum of 8 weeks to a maximum of 78 weeks; weekly intervention frequency varied from 1 to 7 sessions; and single-session duration ranged from 30 to 70 min, with most sessions lasting 60 min. Most studies implemented regular, cyclical intervention plans with on-site organization and guidance provided by exercise instructors, health coaches, or program coordinators. The majority of studies employed an intervention protocol consisting of 2–3 sessions per week, each lasting 40–60 min.

**Table 2:** Characteristics of included trials.

Trial ID	Setting	Duration	Session (Min)	Sessions per week	Control arm
Kadri et al. (30)	S Q G	78 weeks	50 min	2	PET
Cho et al. (21)	S Q G	16 weeks	60 min	5	WLC
Roh et al. (31)	S Q G	16 weeks	60 min	1	PET
Yun et al. (32)	S Q G	8 weeks	30 min	3	Read.
Cho et al. (33)	S NR NR	16 weeks	60 min	5	PET
Lakes et al. (34)	S Q G	36 weeks	45 min	2	PET
Bae et al. (25)	S Q G	16 weeks	60 min	1	LAC PET
Cho et al. (35)	NR NR NR	16 weeks	70 min	5	NT
Moon et al. (36)	NR NR NR	12 weeks	60 min	3	WLC
Jung et al. (37)	S Q G	12 weeks	60 min	3	NT
Kim et al. (38)	S Q G	24 weeks	60 min	3	NT
Kang (39)	S Q G	18 weeks	45 min	2	PET
Wang (40)	S Q G	12 weeks	40 min	7	PET
Zhang et al. (41)	S Q G	15 weeks	45 min	2	PET

S, Supervised; Q, Qualified coach; G, group; NR, No report; PET, Physical Education Training; WLC, Waitlist Control; LAC, Liberal Arts Courses; NT, No training.

### 3.4 Risk of bias

**Figure 2:**
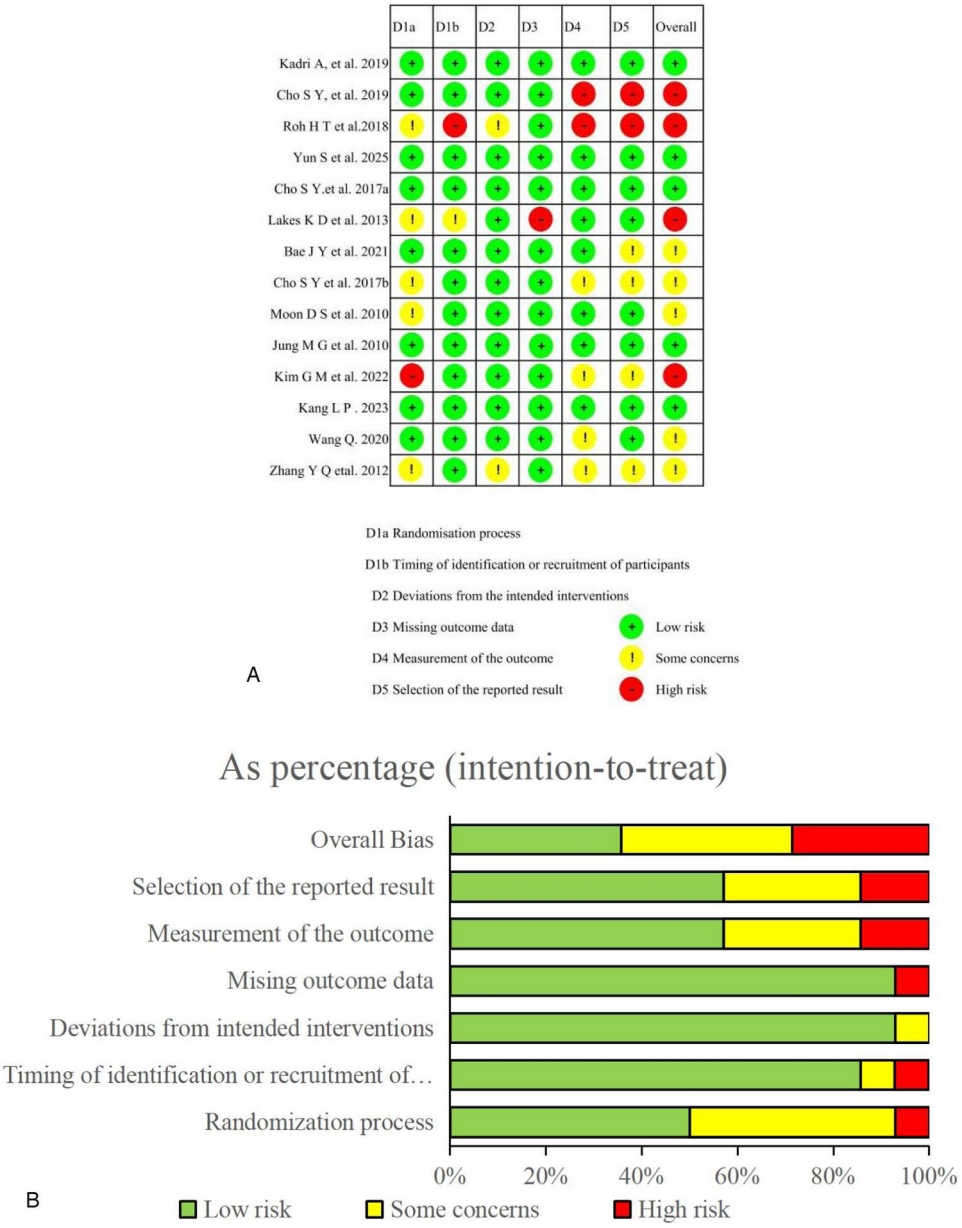
**(A)** risk of bias ratings. **(B)** Risk of bias graph.

The Cochrane RoB 2.0 tool was used to assess the risk of bias in all 14 randomized controlled trials included in this meta-analysis, following the intention-to-treat principle. In accordance with the Cochrane Handbook for Systematic Reviews, each domain and the overall risk of bias were rated as “low risk,” “some concerns,” or “high risk.” Among the 14 studies, five were assessed as having a low overall risk of bias (30, 32, 33, 37, 39), five had some concerns (25, 35, 36, 40, 41), and four were rated as high risk (21, 31, 34, 38). Specifically, eight studies were rated as low risk for bias in randomization (21, 25, 30, 32, 33, 37, 39, 40). Five studies were rated as having some concerns due to insufficient reporting of allocation concealment or sequence generation methods (31, 34–36, 41), and one study was rated as high risk (38) due to incomplete process reporting. Regarding bias in intervention implementation, twelve studies were rated as low risk (21, 25, 30, 32–40), indicating high adherence and stable experimental processes. Data completeness was generally good across studies. Thirteen studies were rated as low risk for missing outcome data (21, 25, 30–33, 35–41), suggesting that most interventions were implemented as planned, with successful data collection and follow-up completion. Only one study (34) showed potential data loss. For outcome measurement bias, eight studies were rated as low risk (25, 30, 32–34, 36, 37), whereas two were rated as high risk due to the absence of assessor blinding or reliance solely on subjective scales (21, 31). Additionally, four studies were classified as having some concerns regarding outcome reporting bias due to the absence of pre-registration or incomplete descriptions of outcome measures (35, 38, 40, 41). Notably, outcome reporting bias was the most prevalent concern. Only eight studies provided complete trial registration information or predefined statistical analysis plans. The remaining studies did not clearly report pre-specified outcomes or analysis strategies, leading to moderate or high risk assessments and suggesting potential selective outcome reporting (see Figures 2A,B for details). Publication bias was evaluated using Begg's rank correlation and Egger's regression tests. For depressive symptoms, Begg's test returned *p* = 0.108, whereas Egger's test indicated *p* = 0.074, showed no evidence of publication bias, with funnel plots displaying overall symmetry (Supplementary S3), suggesting possible small-study effects; thus, results should be interpreted cautiously. For cognitive outcomes, Begg's test (*Z* = 0.39, *p* = 0.755) and Egger's test (*p* = 0.480) showed no evidence of publication bias, with funnel plots displaying overall symmetry (Supplementary S4).

### 3.5 Overall intervention effect

#### 3.5.1 Comparison of Taekwondo training group and control group

**Figure 3:**
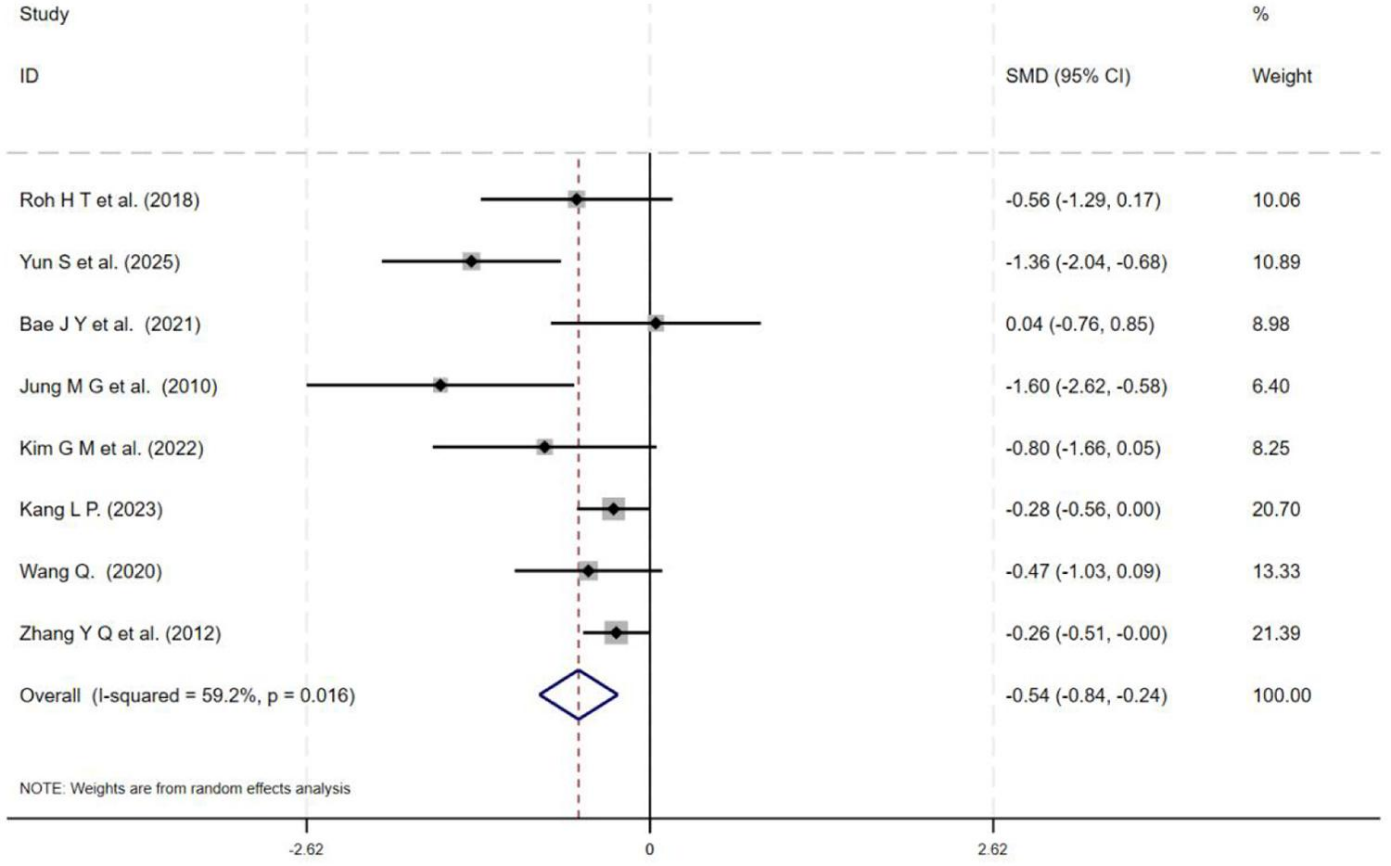
Forest plot of taekwondo intervention on depressive symptoms.

**Figure 4:**
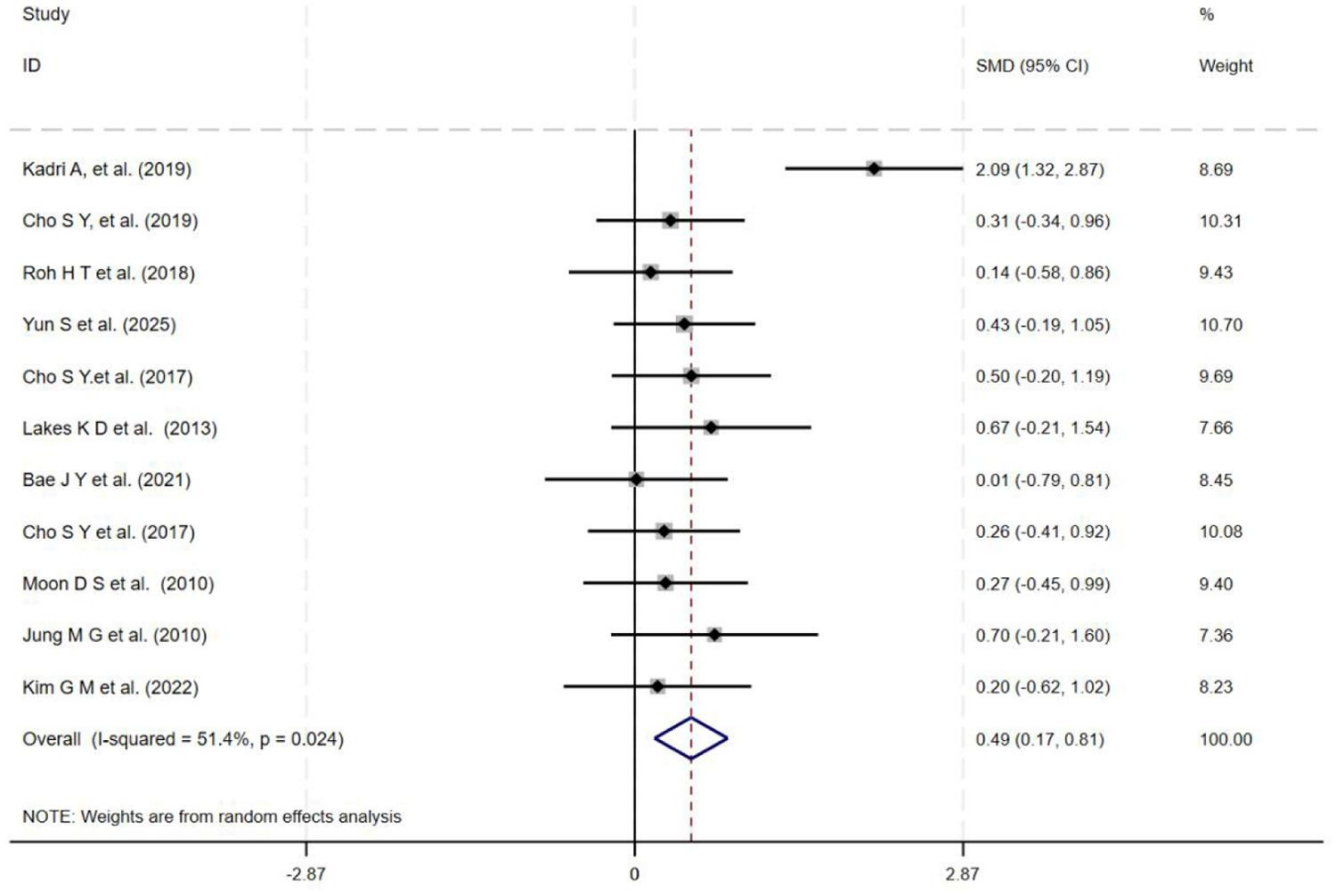
Forest plot of taekwondo intervention on cognitive function.

This meta-analysis included 14 studies. Given the high degree of heterogeneity among studies (*I*^2^ > 50%), a random-effects model was employed to analyze the effects of Taekwondo intervention on depression and cognitive function across the 14 studies using SMD. The results indicate that Taekwondo training has a statistically significant positive effect on improving depression and cognitive function. For depressive symptoms, eight studies involving 643 participants were included, yielding a pooled effect size of SMD = −0.54 (95% CI: −0.84 to −0.24; *I*^2^ = 59.2%; Figure 3). For cognitive function, 11 studies (*n* = 335) were analyzed, demonstrating a significant effect (SMD = 0.49; 95% CI: 0.17–0.81; *I*^2^ = 51.4%; Figure 4). One trial (30) reported a markedly larger effect size than the other included studies. Accordingly, its influence on the pooled estimate was examined. When this trial was excluded, the cognitive effect remained statistically significant (SMD = 0.34, 95% CI: 0.11–0.57), and heterogeneity decreased to *I*^2^ = 0.0%. Because the direction and statistical significance of the effect were unchanged, the primary results presented in the main text are based on all 11 trials. The corresponding forest plot excluding this outlier is provided in Supplementary S7. The negative sign of the effect size indicates that the intervention group showed greater improvement in symptom-related outcomes compared to the control group. Notably, effect sizes in some individual studies approached zero or had confidence intervals that encompassed zero, potentially due to insufficient intervention duration or small sample sizes in the included trials.

#### 3.5.2 Regression analysis

Given that covariates such as intervention duration, frequency, session length, and gender may influence the effectiveness of Taekwondo in improving depression and cognitive function, this study conducted subgroup regression analyses to investigate whether these factors contribute to effect size heterogeneity. Tables 3, 4 present the results of the covariate regression analyses for depression and cognitive function. Regarding depression, the duration of the Taekwondo intervention did not significantly moderate the outcome (*β* = 0.065, 95% CI = −0.922 to 1.052, *p* = 0.847). Similarly, the number of sessions per intervention (*β* = −0.710, 95% CI = −2.074 to 0.653, *p* = 0.196), the duration of each session (*β* = −0.109, 95% CI = −0.934 to 0.717, *p* = 0.703), and participant gender ratio (*β* = 0.145, 95% CI = −1.159 to 1.449, *p* = 0.816) were not significantly associated with effect size. Regarding cognitive function, neither the duration of the Taekwondo intervention duration (*β* = 0.214, 95% CI = −0.314 to 0.742, *p* = 0.360), nor the number of intervention sessions per week (*β* = −0.175, 95% CI = −1.273 to 1.624, *p* = 0.777), session duration (*β* = −0.905, 95% CI = −2.038 to 2.283, *p* = 0.099), nor participant gender ratio (*β* = 0.167, 95% CI = −1.114 to 1.449, *p* = 0.760) showed a significant effect on cognitive outcomes.

**Table 3:** Regression analysis of taekwondo intervention for depression.

_ES	Coefficient	Std. err.	t	*P* > |t|	[95% conf. interval]
Duration (weeks)	0.065	0.310	0.21	0.847	−0.922 to 1.052
Frequency (week)	−0.710	0.429	−1.66	0.196	−2.074 to 0.653
Time (min)	−0.109	0.259	−0.42	0.703	−0.934 to .717
Gender	0.145	0.410	0.35	0.747	−1.160 to 1.449
_cons	0.295	1.160	0.25	0.816	−3.398 to 3.989

**Table 4:** Regression analysis of taekwondo intervention on cognitive function.

_ES	Coefficient	Std. err.	t	*P* > |t|	[95% conf. interval]
Duration (weeks)	0.214	0.216	0.99	0.360	−0.314 to 0.742
Frequency (week)	0.175	0.592	0.30	0.777	−1.273 to 1.624
Time (min)	−0.905	0.463	−1.95	0.099	−2.038 to 0.228
Gender	0.167	0.524	0.32	0.760	−1.114 to 1.449
_cons	1.121	0.831	1.35	0.226	−0.911 to 3.153

#### 3.5.3 Subgroup analysis

To further explore sources of heterogeneity, four subgroup variables were defined based on evidence from prior meta-analyses: intervention duration (weeks), weekly training frequency, single-session duration (minutes), and participant gender ratio. Subgroup analyses were conducted using random-effects models, reporting pooled effect sizes (SMD), confidence intervals, and measures of heterogeneity. It should be noted that subgroup analyses may vary in the number of included studies, as some research reported only one of the two outcomes (depression or cognitive function). The criteria for subgroup classification and corresponding data are provided in Tables 5, 6. For depression, interventions with ≥3 sessions per week (SMD = −0.921, 95% CI = −1.282 to −0.559, *p* = 0.000, *I*^2^ = 48.6%) and a duration of ≤ 12 weeks (SMD = −1.069, 95% CI = −1.785 to −0.353, *p* = 0.003, *I*^2^ = 65.2%) demonstrated significant reduction in depressive symptoms, suggesting that high-intensity, compact training schedules are crucial for alleviating depressive mood. Meanwhile, moderate duration (41—59 min) (SMD = −0.266, 95% CI = −0.453 to −0.079, *p* = 0.005, *I*^2^ = 0.0%) and moderate duration (13–16 weeks; SMD = −0.261, 95% CI = −0.490 to −0.033, *p* = 0.025, *I*^2^ = 0.0%) demonstrated high consistency despite smaller effect sizes, confirming their stable efficacy. Furthermore, intervention effects showed greater robustness in samples with ≤50% female participants (SMD = −0.440, 95% CI = −0.727 to −0.245, *p* = 0.003, *I*^2^ = 48.5%), suggesting that Taekwondo interventions may offer greater benefits for improving depression in women. For cognitive function, interventions with ≥3 sessions per week (SMD = 0.354, 95% CI = 0.057 to 0.651, *p* = 0.019, *I*^2^ = 0.0%) and sessions lasting > 50 min (SMD = 0.290, 95% CI = 0.031 to 0.550, *p* = 0.028, *I*^2^ = 0.0%) with a duration ≤12 weeks (SMD = 0.435, 95% CI = 0.018 to 0.852, *p* = 0.041, *I*^2^ = 0.0%) demonstrated moderate and consistent positive effects, suggesting that sustained and time-intensive training is an effective approach for enhancing cognitive function. Notably, single-session interventions ≤50 min (SMD = 0.982, 95% CI = 0.558 to 1.407, *p* = 0.045, *I*^2^ = 82.3%) exhibited large effect sizes, but were highly heterogeneous and unstable. Gender subgroup analysis revealed more stable effects in groups with ≥50% female participants (SMD = 0.379, 95% CI = 0.047 to 0.710, *p* = 0.025, *I*^2^ = 0.0%), while larger effect sizes but greater heterogeneity were observed in groups with <50% female participants (SMD = 0.591, 95% CI = 0.006 to 1.176, *p* = 0.048, *I*^2^ = 73.7%), suggesting that gender composition may influence the stability of intervention outcomes. Meta-regression within a random-effects framework revealed that intervention duration, training frequency, session length, and gender ratio did not significantly account for variability in effect sizes across studies. The pooled Wald *F*-test (*F* = 1.53, *p* = 0.304) indicated that although numerical differences existed among subgroups, these were insufficient to support reliable subgroup-specific moderation effects, and likely reflected random variation.

**Table 5:** Subgroup analysis of taekwondo intervention for depression,.

Group	Sub-Group	*K*	*N*	SMD	95% CI	*p*	*I* ^2^
Duration (weeks)	≤12	3	111	−1.069	−1.785 to −0.353	0.003	65.2%
	13 ≤ 16	3	309	−0.261	−0.490 to −0.033	0.025	0.00%
	>16	2	223	−0.378	−0.783 to 0.027	0.067	23.8%
Frequency (week)	<3	4	509	−0.268	−0.445 to −0.091	0.003	0.00%
	≥3	4	134	−0.921	−1.282 to −0.559	0.000	48.6%
Time (min)	≤40	2	91	−0.893	−1.765 to −0.020	0.045	74.4%
	41 ≤ 59	2	455	−0.266	−0.453 to −0.079	0.005	0.00%
	>59	4	97	−0.674	−1.287 to −0.060	0.032	52.9%
Gender	Female < 50	3	95	−0.646	−1.445 to 0.154	0.113	71.7%
	Female ≥ 50	5	548	−0.440	−0.727 to −0.245	0.003	48.5%

**Table 6:** Subgroup analysis of taekwondo intervention on cognitive function.

Group	Sub-Group	*K*	*N*	SMD	95% CI	*p*	*I* ^2^
Duration (weeks)	≤12	3	91	0.435	0.018 to 0.852	0.041	0.00%
	13 ≤ 16	5	159	0.259	−0.054 to 0.572	0.105	0.00%
	>16	3	85	0.996	−0.158 to 2.150	0.091	83.0%
Frequency (week)	<3	5	157	0.664	−0.049 to 1.377	0.068	77.8%
	≥3	6	178	0.354	0.057 to 0.651	0.019	0.00%
Time (min)	30 ≤ 50	3	103	0.982	0.558 to 1.407	0.045	82.3%
	>50	8	232	0.290	0.031 to 0.550	0.028	0.00%
Gender	Female < 50	6	192	0.591	0.006 to 1.176	0.048	73.7%
	Female ≥ 50	5	143	0.379	0.047 to 0.710	0.025	0.00%

#### 3.5.4 Certainty of evidence

Using the GRADE approach, the overall certainty of evidence for both primary outcomes was rated as moderate (Supplementary S8). For depressive symptoms, the body of evidence was downgraded one level for risk of bias, as several trials had high or unclear risk in more than one methodological domain, and one level for indirectness because of population heterogeneity across age groups and clinical characteristics. However, the magnitude of the pooled effect and the consistency of direction across studies supported an upgrade for strong association, resulting in an overall rating of moderate certainty. A similar pattern was observed for cognitive outcomes, where limitations in study quality and indirectness were offset in part by a moderate effect size, leading again to a moderate-certainty rating.

## 4 Discussion

A systematic review and meta-analysis were conducted to comprehensively examine existing research on the effects of Taekwondo intervention on depression and cognitive function. The aim was to reveal the potential mechanisms underlying Taekwondo's impact on depression and cognitive function across different age groups and to propose scientifically effective Taekwondo intervention protocols. The findings indicate that Taekwondo training significantly improves depressive symptoms (SMD = −0.54; 95% CI = −0.84 to −0.24; *I*^2^ = 59.2%) and enhances cognitive function (SMD = 0.49; 95% CI = 0.17–0.81; *I*^2^ = 51.4%). These findings confirm the dual health benefits of Taekwondo as an integrated mind-body exercise intervention.

Our findings are consistent with existing evidence demonstrating that Taekwondo alleviates depressive symptoms and enhances cognitive function (42–45). By simultaneously examining depressive symptoms and cognitive outcomes, this review addresses limitations of prior work that focused exclusively on single domains, thereby offering a more comprehensive evaluation of Taekwondo's therapeutic potential. A recent meta-analysis by Gao et al. (46), which included 15 studies, reported significant reductions in depressive symptoms among older women (ES = −0.635, *p* < 0.001), findings that closely align with the effect size estimated in our review. Differences in the number of included studies primarily arose from the more stringent eligibility criteria applied here, as the present review restricted inclusion to peer-reviewed journal articles and randomized controlled trials. Notably, Gao et al. reported substantial heterogeneity (*I*^2^ = 85.8%), whereas the heterogeneity observed in our review was comparatively lower (*I*^2^ = 59.2%), although still moderate to high, suggesting variability in intervention protocols, outcome measures, and study quality (28). Similarly, Ben et al. (47) found an effect size of SMD = −0.45 (*I*^2^ = 71.37%) for general physical activity interventions targeting childhood and adolescent depression. The slightly larger effect observed for Taekwondo in the present review (SMD = −0.54, *I*^2^ = 59.2%) suggests that Taekwondo may confer additional psychological benefits beyond those afforded by physical activity alone. From a self-determination theory perspective, Taekwondo's structured training environment may support the fulfilment of autonomy, competence, and relatedness needs, thereby promoting intrinsic motivation, psychological resilience, and mental well-being (48). Its training principles also foster a strong sense of competence and belonging (49), contributing to a “mind–body unity” process in which external demands become internalised as self-driven behavioural goals. Regarding cognitive function, Zou et al.'s systematic review of 18 studies (50) reported that Taekwondo training enhances cognitive development and inhibitory control in younger populations and attenuates cognitive decline in middle-aged and older adults. However, several studies, such as Bae (25) and Roh et al. (31), did not detect significant cognitive improvements, possibly due to insufficient training frequency. Although previous reviews suggested that Taekwondo benefits mood and cognition in older adults (51, 52), the widespread use of quasi-experimental or observational designs limits the causal inferences that can be drawn (53). By restricting inclusion to randomized controlled trials, the present review provides more robust evidence, but it also narrows the available evidence base and may limit the generalizability and broad applicability of the findings compared with a synthesis that additionally includes high-quality quasi-experimental studies. To further examine potential mechanisms, a narrative theoretical analysis was conducted, and future directions were proposed based on patterns observed in the current literature.

To further explore potential sources of substantial heterogeneity in intervention effects, subgroup analyses were performed. However, because each subgroup contained relatively few studies and heterogeneity persisted, these findings should be interpreted with caution to avoid overgeneralising causal inferences. Short-term interventions (≤12 weeks) yielded the largest improvements in depressive symptoms, whereas long-term interventions produced smaller effect sizes but demonstrated lower heterogeneity. This pattern does not suggest that long-term training is ineffective, but may instead reflect declining adherence or psychological fatigue over extended intervention periods (54, 55). This underscores the need to prioritise sustained motivation and engagement in practical applications and further supports the effectiveness of short-term interventions in regulating depressive symptoms. This is consistent with prior evidence, such as the meta-analysis by Wang et al. showing that an 8-week exercise programme significantly reduced depressive symptoms (SMD = −0.82) (54), and the analysis by Chen et al. reporting greater benefits for interventions lasting fewer than 12 weeks (SMD = −0.76, *p* < 0.01) (56). In relation to cognitive outcomes, long-term interventions (>16 weeks) generated relatively large effect sizes (SMD = 0.996), a finding supported by previous research. Structured exercise may elicit early cognitive gains through mechanisms such as enhanced neuroplasticity, increased secretion of brain-derived neurotrophic factor (BDNF), and improved cerebral blood flow and cognitive arousal (33, 57). Nevertheless, the marked heterogeneity observed for long-term interventions (*I*^2^ = 83.0%) suggests substantial inter-individual variability, potentially attributable to differences in baseline cognitive functioning across diverse populations. With respect to intervention frequency, training at least three times per week yielded significant improvements in both cognitive function (SMD = 0.354) and depressive symptoms (SMD = −0.921). This corresponds to a dose–response pattern widely reported in exercise psychology (58) and aligns with existing evidence (43, 55, 59). However, given the limited number of studies in each subgroup, the influence of residual confounding cannot be excluded, highlighting the need for rigorously designed dose–response trials. Analyses of single-session duration indicated that 30–50-minute sessions produced moderate and stable benefits for both depressive symptoms and cognitive function, consistent with prior findings. Neuroimaging evidence suggests that moderate-duration exercise enhances activation in the prefrontal cortex and hippocampus, thereby supporting emotional regulation and memory processing (60, 61). Regarding gender, evidence suggests that female participants derive more pronounced mental health benefits from Taekwondo training, whereas male participants tend to exhibit stronger cognitive gains. Although these trends are supported by existing literature and are compatible with our findings (62–64), they may be partly attributable to gender-related differences in brain structural connectivity. Given the significant gender imbalance within the included studies, this observation should be interpreted as a preliminary hypothesis rather than a definitive conclusion (65). Overall, moderate-intensity taekwondo interventions (≥3 sessions per week, 30–50 min per session, with a duration of >12 weeks) have been demonstrated to significantly improve depressive symptoms and cognitive function. This finding is consistent with existing research, which indicates that moderate-intensity interventions can effectively enhance mental health and cognitive function (66). For instance, Carta et al. reported that 12 weeks of moderate-intensity exercise, with a minimum of 30 min per week, led to significant improvements in overall cognitive performance (such as processing speed and memory) in older adults (67). Furthermore, a recent systematic review found that exercise interventions, including those of moderate intensity, yielded moderate improvements in depressive symptoms, highlighting exercise as a crucial adjunct strategy for mental health (68).

## 5 Limitations of the study

Although this study synthesised 14 randomised controlled trials and systematically quantified the effects of Taekwondo training on depressive symptoms and cognitive function across diverse age groups, several limitations should be acknowledged. First, the absence of medium- to long-term follow-up assessments in most trials restricts understanding of the durability of intervention effects and prevents evaluation of potential rebound phenomena after training cessation. Second, substantial heterogeneity existed in intervention protocols and outcome assessment tools. Intervention durations ranged from 8 to 78 weeks, weekly frequencies from 1 to 7 sessions, and single-session durations from 30 to 70 min. Depression was measured using tools such as the BDI-II, K-GDS, and SCL-90, while cognitive outcomes were assessed using the Stroop test, K-MMSE, and other measures. Although standardised effect-size pooling and subgroup analyses partially mitigated these variations, they may still influence the precision of pooled estimates and limit cross-study comparability. Third, sample compositions were both small and diverse, encompassing children, adolescents, adults, older adults, and special populations such as individuals with ADHD. Given the pronounced differences in neurodevelopment, cognitive baselines, and emotion-regulation mechanisms across these groups, the underlying pathways through which Taekwondo exerts its effects may differ, thereby limiting the generalisability of aggregated results. Future research should employ high-quality RCTs targeting distinct demographic or clinical populations to determine whether intervention mechanisms remain consistent across age and developmental groups. In addition, by restricting inclusion to randomised controlled trials, we may have excluded potentially informative quasi-experimental and observational studies that could have increased the number and diversity of eligible studies; while this design restriction enhances internal validity, it further constrains the external validity and real-world generalisability of our findings. Fourth, potential cultural bias must be considered. Existing evidence is predominantly derived from East Asian regions—particularly China and South Korea—with minimal representation from Western countries and none from South America, Africa, or Oceania. Given the cultural specificity of Taekwondo as an East Asian martial art, its acceptance, delivery formats, and psychological effects may vary across cultural contexts, constraining the global applicability of the findings. Finally, although subgroup analyses were conducted to explore sources of heterogeneity, the limited number of studies within several subgroups means that these results should be regarded as exploratory rather than definitive.

Despite these limitations, this meta-analysis demonstrates several notable strengths. First, the study restricted inclusion to randomised controlled trials published in peer-reviewed journals, thereby enhancing methodological rigour and reducing risks of bias commonly associated with quasi-experimental or observational designs. Second, unlike prior reviews focusing exclusively on single psychological outcomes, this study concurrently evaluated depressive symptoms and cognitive function, resulting in a more comprehensive assessment of Taekwondo's mental-health benefits. Finally, through subgroup analyses and meta-regression, the study provides valuable exploratory insights into how intervention duration, training frequency, session length, and gender composition may influence intervention efficacy. These findings provide an essential foundation for optimising future Taekwondo-based intervention protocols and guiding the development of tailored clinical or community programmes.

Future research should prioritise multicentre, large-sample, long-term follow-up trials implemented under standardised intervention protocols. It should also evaluate the stability of intervention effects across diverse cultural contexts to produce more reliable, mechanism-informed, and globally generalisable scientific evidence. Such work will offer stronger evidence-based support for Taekwondo as an adjunctive intervention for depression and cognitive impairment.

## 6 Conclusions

On the basis of moderate-certainty evidence as rated by GRADE, this meta-analysis indicates that Taekwondo training demonstrates a moderate-to-large effect size in improving depressive symptoms and enhancing cognitive function. Specifically, Taekwondo training significantly improves depressive symptoms, with the most pronounced effects observed in interventions conducted ≥3 times per week, lasting ≤40 min per session, and continuing for ≤12 weeks. In contrast, the enhancement of cognitive function through Taekwondo training is more pronounced in moderate-intensity interventions conducted ≥3 times per week, lasting 30–50 min per session, and continuing for >16 weeks. Furthermore, the research indicates that female participants derive more pronounced psychological and cognitive benefits from Taekwondo training. Subgroup analyses suggest that variables such as intervention frequency, duration, and intensity play crucial moderating roles in treatment outcomes. However, it should be noted that the number of included studies is limited, sample sizes are generally small, and some studies exhibit methodological flaws, including non-standardized randomization procedures and a lack of pre-registration of analysis plans. The robustness and broad applicability of the current findings require further validation through future randomized controlled trials with larger sample sizes and higher methodological quality.
