# Supplementary material for: The effects of taekwondo on depression symptoms and cognitive function: a systematic review and meta-analysis
Source: Front Sports Act Living. 2026 Jan 15;7:1735531. doi: 10.3389/fspor.2025.1735531 (PMC12852433; doi:10.3389/fspor.2025.1735531)
Supplement: Supplementary file 1 [file Datasheet1.docx]

# Supplementary S1: PRISMA checklist

| **Section and Topic** | **Item** | **Checklist item** | **Location where item is reported** |
| --- | --- | --- | --- |
| **TITLE** | | |  |
| Title | 1 | The Effects of Taekwondo on Depressive Symptoms and Cognitive Function: A Systematic Review and Meta-Analysis | The Effects of Taekwondo on Depressive Symptoms and Cognitive Function: A Systematic Review and Meta-Analysis |
| **ABSTRACT** | | |  |
| Abstract | 2 | See the PRISMA 2020 for Abstracts checklist. | Structured Abstract section（Background, Methods, Results, Conclusion） |
| **INTRODUCTION** | | |  |
| Rationale | 3 | Describe the rationale for the review in the context of existing knowledge. | Introduction, Paragraphs 1–3 |
| Objectives | 4 | Provide an explicit statement of the objective(s) or question(s) the review addresses. | Introduction, final paragraph |
| **METHODS** | | |  |
| Eligibility criteria | 5 | Specify the inclusion and exclusion criteria for the review and how studies were grouped for the syntheses. | Methods 2.2 (PICOS framework; inclusion/exclusion criteria). |
| Information sources | 6 | Specify all databases, registers, websites, organisations, reference lists and other sources searched or consulted to identify studies. Specify the date when each source was last searched or consulted. | Methods 2.1 Search Strategy (PubMed, Embase, Cochrane, Web of Science Core Collection, Ovid MEDLINE, CNKI, Wanfang; search completed Sept 2025). |
| Search strategy | 7 | Present the full search strategies for all databases, registers and websites, including any filters and limits used. | Methods 2.1; Supplementary 2 |
| Selection process | 8 | Specify the methods used to decide whether a study met the inclusion criteria of the review, including how many reviewers screened each record and each report retrieved, whether they worked independently, and if applicable, details of automation tools used in the process. | Methods 2.2; PRISMA Flow Diagram (two reviewers independently screened; third reviewer resolved disagreements). |
| Data collection process | 9 | Specify the methods used to collect data from reports, including how many reviewers collected data from each report, whether they worked independently, any processes for obtaining or confirming data from study investigators, and if applicable, details of automation tools used in the process. | Methods 2.3 Data Extraction (two reviewers independently extracted data; third reviewer adjudicated disagreements). |
| Data items | 10a | List and define all outcomes for which data were sought. Specify whether all results that were compatible with each outcome domain in each study were sought (e.g. for all measures, time points, analyses), and if not, the methods used to decide which results to collect. | Methods 2.3: depressive symptoms (BDI-II, K-GDS, SCL-90), cognitive outcomes (Stroop, K-MMSE, etc.). |
|  | 10b | List and define all other variables for which data were sought (e.g. participant and intervention characteristics, funding sources). Describe any assumptions made about any missing or unclear information. | Methods 2.3: age, gender, sample size, intervention program, duration, weekly frequency, session length, country, measurement instruments. |
| Study risk of bias assessment | 11 | Specify the methods used to assess risk of bias in the included studies, including details of the tool(s) used, how many reviewers assessed each study and whether they worked independently, and if applicable, details of automation tools used in the process. | Methods 2.4 Risk of Bias Assessment |
| Effect measures | 12 | Specify for each outcome the effect measure(s) (e.g. risk ratio, mean difference) used in the synthesis or presentation of results. | Methods 2.5 Statistical Analysis |
| Synthesis methods | 13a | Describe the processes used to decide which studies were eligible for each synthesis (e.g. tabulating the study intervention characteristics and comparing against the planned groups for each synthesis (item #5)). | Methods 2.2 + PRISMA Flowchart. |
|  | 13b | Describe any methods required to prepare the data for presentation or synthesis, such as handling of missing summary statistics, or data conversions. | Methods 2.5 Statistical Analysis |
|  | 13c | Describe any methods used to tabulate or visually display results of individual studies and syntheses. | Forest plots (Figures 3 and 4); supplementary figures. |
|  | 13d | Describe any methods used to synthesize results and provide a rationale for the choice(s). If meta-analysis was performed, describe the model(s), method(s) to identify the presence and extent of statistical heterogeneity, and software package(s) used. | Methods 2.5 Statistical Analysis |
|  | 13e | Describe any methods used to explore possible causes of heterogeneity among study results (e.g. subgroup analysis, meta-regression). | Subgroup analyses + meta-regression (Methods 2.5; Tables 6 & 7). |
|  | 13f | Describe any sensitivity analyses conducted to assess robustness of the synthesized results. | Methods 2.5 + Results (Supplementary S5 and S6). |
| Reporting bias assessment | 14 | Describe any methods used to assess risk of bias due to missing results in a synthesis (arising from reporting biases). | Methods 2.5: Begg's test, Egger's test; funnel plots (Supplementary S3 and S4). |
| Certainty assessment | 15 | Describe any methods used to assess certainty (or confidence) in the body of evidence for an outcome. | Methods 2.5 |
| **RESULTS** | | |  |
| Study selection | 16a | Describe the results of the search and selection process, from the number of records identified in the search to the number of studies included in the review, ideally using a flow diagram. | 3.1 Studies Selection |
|  | 16b | Cite studies that might appear to meet the inclusion criteria, but which were excluded, and explain why they were excluded. | 3.3 Interventions and Controls |
| Study characteristics | 17 | Cite each included study and present its characteristics. | Table 1 |
| Risk of bias in studies | 18 | Present assessments of risk of bias for each included study. | Risk of Bias Assessment; Figures 2A–2B |
| Results of individual studies | 19 | For all outcomes, present, for each study: (a) summary statistics for each group (where appropriate) and (b) an effect estimate and its precision (e.g. confidence/credible interval), ideally using structured tables or plots. | 3.5.1 Comparison of Taekwondo exercise group and control group |
| Results of syntheses | 20a | For each synthesis, briefly summarise the characteristics and risk of bias among contributing studies. | 3.5.1 Comparison of Taekwondo exercise group and control group |
|  | 20b | Present results of all statistical syntheses conducted. If meta-analysis was done, present for each the summary estimate and its precision (e.g. confidence/credible interval) and measures of statistical heterogeneity. If comparing groups, describe the direction of the effect. | 3.5.1 Comparison of Taekwondo exercise group and control group |
|  | 20c | Present results of all investigations of possible causes of heterogeneity among study results. | 3.5.3 Subgroup Analyses; 3.5.4 Meta-regression |
|  | 20d | Present results of all sensitivity analyses conducted to assess the robustness of the synthesized results. | Sensitivity Analysis; Supplementary5 and S6 |
| Reporting biases | 21 | Present assessments of risk of bias due to missing results (arising from reporting biases) for each synthesis assessed. | 3.4 Risk of bias |
| Certainty of evidence | 22 | Present assessments of certainty (or confidence) in the body of evidence for each outcome assessed. | Methods 2.5 |
| **DISCUSSION** | | |  |
| Discussion | 23a | Provide a general interpretation of the results in the context of other evidence. | Discussion → first three paragraphs |
|  | 23b | Discuss any limitations of the evidence included in the review. | Limitations → first two paragraphs |
|  | 23c | Discuss any limitations of the review processes used. | Limitations → paragraph discussing heterogeneity |
|  | 23d | Discuss implications of the results for practice, policy, and future research. | Discussion → final paragraphs; Limitations → final section |
| **OTHER INFORMATION** | | |  |
| Registration and protocol | 24a | Provide registration information for the review, including register name and registration number, or state that the review was not registered. | Methods → PRISMA Compliance: PROSPERO CRD420251173580 |
|  | 24b | Indicate where the review protocol can be accessed, or state that a protocol was not prepared. | Methods → Protocol Registration |
|  | 24c | Describe and explain any amendments to information provided at registration or in the protocol. | Methods → brief statement under Protocol |
| Support | 25 | Describe sources of financial or non-financial support for the review, and the role of the funders or sponsors in the review. | Funding Statement section |
| Competing interests | 26 | Declare any competing interests of review authors. | Conflict of Interest section |
| Availability of data, code and other materials | 27 | Report which of the following are publicly available and where they can be found: template data collection forms; data extracted from included studies; data used for all analyses; analytic code; any other materials used in the review. | Data Availability Statement: “All data and materials used in this review are available upon reasonable request.” |

# Supplementary Information S2: Search Strategy

## PubMed（1011）

| **Search number** | **Query** | **Sort By** | **Filters** | **Search Details** | **Results** | **Time** | **Date** |
| --- | --- | --- | --- | --- | --- | --- | --- |
| 12 | (((((((("Executive Function"[Mesh]) OR "Attention"[Mesh]) OR "Memory"[Mesh]) OR "Recognition, Psychology"[Mesh]) OR "Cognition"[Mesh]) OR "Cognitive Flexibility"[Mesh]) OR (((((((((((((((((((((((((((((((((Focus of Attention[Title/Abstract]) OR (Attention Focus[Title/Abstract])) OR (Mental Concentration[Title/Abstract])) OR (Concentration, Mental[Title/Abstract])) OR (Social Attention[Title/Abstract])) OR (Attention, Social[Title/Abstract])) OR (Selective Attention[Title/Abstract])) OR (Attention, Selective[Title/Abstract])) OR (Executive Functions[Title/Abstract])) OR (Function, Executive[Title/Abstract])) OR (Functions, Executive[Title/Abstract])) OR (Executive Control[Title/Abstract])) OR (Executive Controls[Title/Abstract])) OR (Psychology Recognition[Title/Abstract])) OR (Psychological Recognition[Title/Abstract])) OR (Recognition, Psychological[Title/Abstract])) OR (Recognition (Psychology[Title/Abstract]))) OR (Familiarity[Title/Abstract])) OR (Cognitions[Title/Abstract])) OR (Cognitive Function[Title/Abstract])) OR (Cognitive Functions[Title/Abstract])) OR (Function, Cognitive[Title/Abstract])) OR (Functions, Cognitive[Title/Abstract])) OR (Insight[Title/Abstract])) OR (Insights[Title/Abstract])) OR (Cognitive Flexibilities[Title/Abstract])) OR (Cognitive Flexibilities[Title/Abstract])) OR (Flexibility, Cognitive[Title/Abstract])) OR (Flexibility, Cognitive[Title/Abstract])) OR (Cognitive Inflexibilities[Title/Abstract])) OR (Inflexibilities, Cognitive[Title/Abstract])) OR (Inflexibility, Cognitive[Title/Abstract])))) OR ((((("Mental Health"[Mesh]) OR "Depression"[Mesh]) OR "Anxiety"[Mesh]) OR "Emotions"[Mesh]) OR ((((((((((((((((((((((((Health, Mental[Title/Abstract]) OR (Mental Hygiene[Title/Abstract])) OR (Hygiene, Mental[Title/Abstract])) OR (Depressive Symptoms[Title/Abstract])) OR (Depressive Symptom[Title/Abstract])) OR (Symptom, Depressive[Title/Abstract])) OR (Emotional Depression[Title/Abstract])) OR (Depression, Emotional[Title/Abstract])) OR (Angst[Title/Abstract])) OR (Nervousness[Title/Abstract])) OR (Hypervigilance[Title/Abstract])) OR (Hypervigilance[Title/Abstract])) OR (Anxieties, Social[Title/Abstract])) OR (Anxiety, Social[Title/Abstract])) OR (Social Anxieties[Title/Abstract])) OR (Anxiousness[Title/Abstract])) OR (Emotion[Title/Abstract])) OR (Feelings[Title/Abstract])) OR (Feeling[Title/Abstract])) OR (Regret[Title/Abstract])) OR (Regrets[Title/Abstract])) OR (Happinesses[Title/Abstract])) OR (Joy[Title/Abstract])) OR (Angers[Title/Abstract])))) AND ((("Martial Arts"[Mesh]) OR "Tai Ji"[Mesh]) OR ((((((((((((((((((((((Arts, Martial[Title/Abstract]) OR (Judo[Title/Abstract])) OR (Karate[Title/Abstract])) OR (Kung Fu[Title/Abstract])) OR (Gongfu[Title/Abstract])) OR (Gong Fu[Title/Abstract])) OR (Fu, Gong[Title/Abstract])) OR (Tae Kwon Do[Title/Abstract])) OR (Wushu[Title/Abstract])) OR (Hap Ki Do[Title/Abstract])) OR (Aikid[Title/Abstract])) OR (Jujitsu[Title/Abstract])) OR (Tai-ji[Title/Abstract])) OR (Tai Chi[Title/Abstract])) OR (Chi, Tai[Title/Abstract])) OR (Tai Chi Chuan[Title/Abstract])) OR (Taiji[Title/Abstract])) OR (Taijiqun[Title/Abstract])) OR (T'ai Chi[Title/Abstract])) OR (Tai Ji Quan[Title/Abstract])) OR (Ji Quan, Tai[Title/Abstract])) OR (Quan, Tai Ji[Title/Abstract]))) | Publication Date | from 1000/1/1 - 2025/9/21 | (("Executive Function"[MeSH Terms] OR "Attention"[MeSH Terms] OR "Memory"[MeSH Terms] OR "recognition, psychology"[MeSH Terms] OR "Cognition"[MeSH Terms] OR "Cognitive Flexibility"[MeSH Terms] OR ("focus of attention"[Title/Abstract] OR "attention focus"[Title/Abstract] OR "mental concentration"[Title/Abstract] OR "concentration mental"[Title/Abstract] OR "social attention"[Title/Abstract] OR "attention social"[Title/Abstract] OR "selective attention"[Title/Abstract] OR "attention selective"[Title/Abstract] OR "executive functions"[Title/Abstract] OR "function executive"[Title/Abstract] OR "functions executive"[Title/Abstract] OR "executive control"[Title/Abstract] OR "executive controls"[Title/Abstract] OR "psychology recognition"[Title/Abstract] OR "psychological recognition"[Title/Abstract] OR "recognition psychological"[Title/Abstract] OR (("recognition, psychology"[MeSH Terms] OR ("Recognition"[All Fields] AND "Psychology"[All Fields]) OR "psychology recognition"[All Fields] OR "Recognition"[All Fields] OR "recognitions"[All Fields]) AND "Psychology"[Title/Abstract]) OR "Familiarity"[Title/Abstract] OR "Cognitions"[Title/Abstract] OR "cognitive function"[Title/Abstract] OR "cognitive functions"[Title/Abstract] OR "function cognitive"[Title/Abstract] OR "functions cognitive"[Title/Abstract] OR "Insight"[Title/Abstract] OR "Insights"[Title/Abstract] OR "cognitive flexibilities"[Title/Abstract] OR "cognitive flexibilities"[Title/Abstract] OR "flexibility cognitive"[Title/Abstract] OR "flexibility cognitive"[Title/Abstract] OR (("Cognition"[MeSH Terms] OR "Cognition"[All Fields] OR "Cognitions"[All Fields] OR "Cognitive"[All Fields] OR "cognitively"[All Fields] OR "cognitives"[All Fields]) AND "Inflexibilities"[Title/Abstract]) OR ("Inflexibilities"[All Fields] AND "Cognitive"[Title/Abstract]) OR "inflexibility cognitive"[Title/Abstract]) OR ("Mental Health"[MeSH Terms] OR "Depression"[MeSH Terms] OR "Anxiety"[MeSH Terms] OR "Emotions"[MeSH Terms] OR ("health mental"[Title/Abstract] OR "mental hygiene"[Title/Abstract] OR "hygiene mental"[Title/Abstract] OR "depressive symptoms"[Title/Abstract] OR "depressive symptom"[Title/Abstract] OR "symptom depressive"[Title/Abstract] OR "emotional depression"[Title/Abstract] OR "depression emotional"[Title/Abstract] OR "Angst"[Title/Abstract] OR "Nervousness"[Title/Abstract] OR "Hypervigilance"[Title/Abstract] OR "Hypervigilance"[Title/Abstract] OR (("Anxiety"[MeSH Terms] OR "Anxiety"[All Fields] OR "Anxieties"[All Fields] OR "anxiety s"[All Fields]) AND "Social"[Title/Abstract]) OR "anxiety social"[Title/Abstract] OR "social anxieties"[Title/Abstract] OR "Anxiousness"[Title/Abstract] OR "Emotion"[Title/Abstract] OR "Feelings"[Title/Abstract] OR "Feeling"[Title/Abstract] OR "Regret"[Title/Abstract] OR "Regrets"[Title/Abstract] OR "Happinesses"[Title/Abstract] OR "Joy"[Title/Abstract] OR "Angers"[Title/Abstract]))) AND ("Martial Arts"[MeSH Terms] OR "tai ji"[MeSH Terms] OR ("arts martial"[Title/Abstract] OR "Judo"[Title/Abstract] OR "Karate"[Title/Abstract] OR "kung fu"[Title/Abstract] OR "Gongfu"[Title/Abstract] OR "gong fu"[Title/Abstract] OR "fu gong"[Title/Abstract] OR "tae kwon do"[Title/Abstract] OR "Wushu"[Title/Abstract] OR ("Hap"[All Fields] AND "ki do"[Title/Abstract]) OR "Jujitsu"[Title/Abstract] OR "tai ji"[Title/Abstract] OR "tai chi"[Title/Abstract] OR "chi tai"[Title/Abstract] OR "tai chi chuan"[Title/Abstract] OR "Taiji"[Title/Abstract] OR "t ai chi"[Title/Abstract] OR "tai ji quan"[Title/Abstract] OR "ji quan tai"[Title/Abstract] OR "quan tai ji"[Title/Abstract]))) AND (1000/1/1:2025/9/20[pdat]) | 1,011 | 23:27:12 | 2025/9/20 |
| 11 | (((((((("Executive Function"[Mesh]) OR "Attention"[Mesh]) OR "Memory"[Mesh]) OR "Recognition, Psychology"[Mesh]) OR "Cognition"[Mesh]) OR "Cognitive Flexibility"[Mesh]) OR (((((((((((((((((((((((((((((((((Focus of Attention[Title/Abstract]) OR (Attention Focus[Title/Abstract])) OR (Mental Concentration[Title/Abstract])) OR (Concentration, Mental[Title/Abstract])) OR (Social Attention[Title/Abstract])) OR (Attention, Social[Title/Abstract])) OR (Selective Attention[Title/Abstract])) OR (Attention, Selective[Title/Abstract])) OR (Executive Functions[Title/Abstract])) OR (Function, Executive[Title/Abstract])) OR (Functions, Executive[Title/Abstract])) OR (Executive Control[Title/Abstract])) OR (Executive Controls[Title/Abstract])) OR (Psychology Recognition[Title/Abstract])) OR (Psychological Recognition[Title/Abstract])) OR (Recognition, Psychological[Title/Abstract])) OR (Recognition (Psychology[Title/Abstract]))) OR (Familiarity[Title/Abstract])) OR (Cognitions[Title/Abstract])) OR (Cognitive Function[Title/Abstract])) OR (Cognitive Functions[Title/Abstract])) OR (Function, Cognitive[Title/Abstract])) OR (Functions, Cognitive[Title/Abstract])) OR (Insight[Title/Abstract])) OR (Insights[Title/Abstract])) OR (Cognitive Flexibilities[Title/Abstract])) OR (Cognitive Flexibilities[Title/Abstract])) OR (Flexibility, Cognitive[Title/Abstract])) OR (Flexibility, Cognitive[Title/Abstract])) OR (Cognitive Inflexibilities[Title/Abstract])) OR (Inflexibilities, Cognitive[Title/Abstract])) OR (Inflexibility, Cognitive[Title/Abstract])))) OR ((((("Mental Health"[Mesh]) OR "Depression"[Mesh]) OR "Anxiety"[Mesh]) OR "Emotions"[Mesh]) OR ((((((((((((((((((((((((Health, Mental[Title/Abstract]) OR (Mental Hygiene[Title/Abstract])) OR (Hygiene, Mental[Title/Abstract])) OR (Depressive Symptoms[Title/Abstract])) OR (Depressive Symptom[Title/Abstract])) OR (Symptom, Depressive[Title/Abstract])) OR (Emotional Depression[Title/Abstract])) OR (Depression, Emotional[Title/Abstract])) OR (Angst[Title/Abstract])) OR (Nervousness[Title/Abstract])) OR (Hypervigilance[Title/Abstract])) OR (Hypervigilance[Title/Abstract])) OR (Anxieties, Social[Title/Abstract])) OR (Anxiety, Social[Title/Abstract])) OR (Social Anxieties[Title/Abstract])) OR (Anxiousness[Title/Abstract])) OR (Emotion[Title/Abstract])) OR (Feelings[Title/Abstract])) OR (Feeling[Title/Abstract])) OR (Regret[Title/Abstract])) OR (Regrets[Title/Abstract])) OR (Happinesses[Title/Abstract])) OR (Joy[Title/Abstract])) OR (Angers[Title/Abstract])))) AND ((("Martial Arts"[Mesh]) OR "Tai Ji"[Mesh]) OR ((((((((((((((((((((((Arts, Martial[Title/Abstract]) OR (Judo[Title/Abstract])) OR (Karate[Title/Abstract])) OR (Kung Fu[Title/Abstract])) OR (Gongfu[Title/Abstract])) OR (Gong Fu[Title/Abstract])) OR (Fu, Gong[Title/Abstract])) OR (Tae Kwon Do[Title/Abstract])) OR (Wushu[Title/Abstract])) OR (Hap Ki Do[Title/Abstract])) OR (Aikid[Title/Abstract])) OR (Jujitsu[Title/Abstract])) OR (Tai-ji[Title/Abstract])) OR (Tai Chi[Title/Abstract])) OR (Chi, Tai[Title/Abstract])) OR (Tai Chi Chuan[Title/Abstract])) OR (Taiji[Title/Abstract])) OR (Taijiqun[Title/Abstract])) OR (T'ai Chi[Title/Abstract])) OR (Tai Ji Quan[Title/Abstract])) OR (Ji Quan, Tai[Title/Abstract])) OR (Quan, Tai Ji[Title/Abstract]))) | Publication Date |  | ("Executive Function"[MeSH Terms] OR "Attention"[MeSH Terms] OR "Memory"[MeSH Terms] OR "recognition, psychology"[MeSH Terms] OR "Cognition"[MeSH Terms] OR "Cognitive Flexibility"[MeSH Terms] OR ("focus of attention"[Title/Abstract] OR "attention focus"[Title/Abstract] OR "mental concentration"[Title/Abstract] OR "concentration mental"[Title/Abstract] OR "social attention"[Title/Abstract] OR "attention social"[Title/Abstract] OR "selective attention"[Title/Abstract] OR "attention selective"[Title/Abstract] OR "executive functions"[Title/Abstract] OR "function executive"[Title/Abstract] OR "functions executive"[Title/Abstract] OR "executive control"[Title/Abstract] OR "executive controls"[Title/Abstract] OR "psychology recognition"[Title/Abstract] OR "psychological recognition"[Title/Abstract] OR "recognition psychological"[Title/Abstract] OR (("recognition, psychology"[MeSH Terms] OR ("Recognition"[All Fields] AND "Psychology"[All Fields]) OR "psychology recognition"[All Fields] OR "Recognition"[All Fields] OR "recognitions"[All Fields]) AND "Psychology"[Title/Abstract]) OR "Familiarity"[Title/Abstract] OR "Cognitions"[Title/Abstract] OR "cognitive function"[Title/Abstract] OR "cognitive functions"[Title/Abstract] OR "function cognitive"[Title/Abstract] OR "functions cognitive"[Title/Abstract] OR "Insight"[Title/Abstract] OR "Insights"[Title/Abstract] OR "cognitive flexibilities"[Title/Abstract] OR "cognitive flexibilities"[Title/Abstract] OR "flexibility cognitive"[Title/Abstract] OR "flexibility cognitive"[Title/Abstract] OR (("Cognition"[MeSH Terms] OR "Cognition"[All Fields] OR "Cognitions"[All Fields] OR "Cognitive"[All Fields] OR "cognitively"[All Fields] OR "cognitives"[All Fields]) AND "Inflexibilities"[Title/Abstract]) OR ("Inflexibilities"[All Fields] AND "Cognitive"[Title/Abstract]) OR "inflexibility cognitive"[Title/Abstract]) OR ("Mental Health"[MeSH Terms] OR "Depression"[MeSH Terms] OR "Anxiety"[MeSH Terms] OR "Emotions"[MeSH Terms] OR ("health mental"[Title/Abstract] OR "mental hygiene"[Title/Abstract] OR "hygiene mental"[Title/Abstract] OR "depressive symptoms"[Title/Abstract] OR "depressive symptom"[Title/Abstract] OR "symptom depressive"[Title/Abstract] OR "emotional depression"[Title/Abstract] OR "depression emotional"[Title/Abstract] OR "Angst"[Title/Abstract] OR "Nervousness"[Title/Abstract] OR "Hypervigilance"[Title/Abstract] OR "Hypervigilance"[Title/Abstract] OR (("Anxiety"[MeSH Terms] OR "Anxiety"[All Fields] OR "Anxieties"[All Fields] OR "anxiety s"[All Fields]) AND "Social"[Title/Abstract]) OR "anxiety social"[Title/Abstract] OR "social anxieties"[Title/Abstract] OR "Anxiousness"[Title/Abstract] OR "Emotion"[Title/Abstract] OR "Feelings"[Title/Abstract] OR "Feeling"[Title/Abstract] OR "Regret"[Title/Abstract] OR "Regrets"[Title/Abstract] OR "Happinesses"[Title/Abstract] OR "Joy"[Title/Abstract] OR "Angers"[Title/Abstract]))) AND ("Martial Arts"[MeSH Terms] OR "tai ji"[MeSH Terms] OR ("arts martial"[Title/Abstract] OR "Judo"[Title/Abstract] OR "Karate"[Title/Abstract] OR "kung fu"[Title/Abstract] OR "Gongfu"[Title/Abstract] OR "gong fu"[Title/Abstract] OR "fu gong"[Title/Abstract] OR "tae kwon do"[Title/Abstract] OR "Wushu"[Title/Abstract] OR ("Hap"[All Fields] AND "ki do"[Title/Abstract]) OR "Jujitsu"[Title/Abstract] OR "tai ji"[Title/Abstract] OR "tai chi"[Title/Abstract] OR "chi tai"[Title/Abstract] OR "tai chi chuan"[Title/Abstract] OR "Taiji"[Title/Abstract] OR "t ai chi"[Title/Abstract] OR "tai ji quan"[Title/Abstract] OR "ji quan tai"[Title/Abstract] OR "quan tai ji"[Title/Abstract])) | 1,011 | 23:25:05 | 2025/9/20 |
| 10 | ((((((("Executive Function"[Mesh]) OR "Attention"[Mesh]) OR "Memory"[Mesh]) OR "Recognition, Psychology"[Mesh]) OR "Cognition"[Mesh]) OR "Cognitive Flexibility"[Mesh]) OR (((((((((((((((((((((((((((((((((Focus of Attention[Title/Abstract]) OR (Attention Focus[Title/Abstract])) OR (Mental Concentration[Title/Abstract])) OR (Concentration, Mental[Title/Abstract])) OR (Social Attention[Title/Abstract])) OR (Attention, Social[Title/Abstract])) OR (Selective Attention[Title/Abstract])) OR (Attention, Selective[Title/Abstract])) OR (Executive Functions[Title/Abstract])) OR (Function, Executive[Title/Abstract])) OR (Functions, Executive[Title/Abstract])) OR (Executive Control[Title/Abstract])) OR (Executive Controls[Title/Abstract])) OR (Psychology Recognition[Title/Abstract])) OR (Psychological Recognition[Title/Abstract])) OR (Recognition, Psychological[Title/Abstract])) OR (Recognition (Psychology[Title/Abstract]))) OR (Familiarity[Title/Abstract])) OR (Cognitions[Title/Abstract])) OR (Cognitive Function[Title/Abstract])) OR (Cognitive Functions[Title/Abstract])) OR (Function, Cognitive[Title/Abstract])) OR (Functions, Cognitive[Title/Abstract])) OR (Insight[Title/Abstract])) OR (Insights[Title/Abstract])) OR (Cognitive Flexibilities[Title/Abstract])) OR (Cognitive Flexibilities[Title/Abstract])) OR (Flexibility, Cognitive[Title/Abstract])) OR (Flexibility, Cognitive[Title/Abstract])) OR (Cognitive Inflexibilities[Title/Abstract])) OR (Inflexibilities, Cognitive[Title/Abstract])) OR (Inflexibility, Cognitive[Title/Abstract])))) OR ((((("Mental Health"[Mesh]) OR "Depression"[Mesh]) OR "Anxiety"[Mesh]) OR "Emotions"[Mesh]) OR ((((((((((((((((((((((((Health, Mental[Title/Abstract]) OR (Mental Hygiene[Title/Abstract])) OR (Hygiene, Mental[Title/Abstract])) OR (Depressive Symptoms[Title/Abstract])) OR (Depressive Symptom[Title/Abstract])) OR (Symptom, Depressive[Title/Abstract])) OR (Emotional Depression[Title/Abstract])) OR (Depression, Emotional[Title/Abstract])) OR (Angst[Title/Abstract])) OR (Nervousness[Title/Abstract])) OR (Hypervigilance[Title/Abstract])) OR (Hypervigilance[Title/Abstract])) OR (Anxieties, Social[Title/Abstract])) OR (Anxiety, Social[Title/Abstract])) OR (Social Anxieties[Title/Abstract])) OR (Anxiousness[Title/Abstract])) OR (Emotion[Title/Abstract])) OR (Feelings[Title/Abstract])) OR (Feeling[Title/Abstract])) OR (Regret[Title/Abstract])) OR (Regrets[Title/Abstract])) OR (Happinesses[Title/Abstract])) OR (Joy[Title/Abstract])) OR (Angers[Title/Abstract]))) | Publication Date |  | "Executive Function"[MeSH Terms] OR "Attention"[MeSH Terms] OR "Memory"[MeSH Terms] OR "recognition, psychology"[MeSH Terms] OR "Cognition"[MeSH Terms] OR "Cognitive Flexibility"[MeSH Terms] OR ("focus of attention"[Title/Abstract] OR "attention focus"[Title/Abstract] OR "mental concentration"[Title/Abstract] OR "concentration mental"[Title/Abstract] OR "social attention"[Title/Abstract] OR "attention social"[Title/Abstract] OR "selective attention"[Title/Abstract] OR "attention selective"[Title/Abstract] OR "executive functions"[Title/Abstract] OR "function executive"[Title/Abstract] OR "functions executive"[Title/Abstract] OR "executive control"[Title/Abstract] OR "executive controls"[Title/Abstract] OR "psychology recognition"[Title/Abstract] OR "psychological recognition"[Title/Abstract] OR "recognition psychological"[Title/Abstract] OR (("recognition, psychology"[MeSH Terms] OR ("Recognition"[All Fields] AND "Psychology"[All Fields]) OR "psychology recognition"[All Fields] OR "Recognition"[All Fields] OR "recognitions"[All Fields]) AND "Psychology"[Title/Abstract]) OR "Familiarity"[Title/Abstract] OR "Cognitions"[Title/Abstract] OR "cognitive function"[Title/Abstract] OR "cognitive functions"[Title/Abstract] OR "function cognitive"[Title/Abstract] OR "functions cognitive"[Title/Abstract] OR "Insight"[Title/Abstract] OR "Insights"[Title/Abstract] OR "cognitive flexibilities"[Title/Abstract] OR "cognitive flexibilities"[Title/Abstract] OR "flexibility cognitive"[Title/Abstract] OR "flexibility cognitive"[Title/Abstract] OR (("Cognition"[MeSH Terms] OR "Cognition"[All Fields] OR "Cognitions"[All Fields] OR "Cognitive"[All Fields] OR "cognitively"[All Fields] OR "cognitives"[All Fields]) AND "Inflexibilities"[Title/Abstract]) OR ("Inflexibilities"[All Fields] AND "Cognitive"[Title/Abstract]) OR "inflexibility cognitive"[Title/Abstract]) OR ("Mental Health"[MeSH Terms] OR "Depression"[MeSH Terms] OR "Anxiety"[MeSH Terms] OR "Emotions"[MeSH Terms] OR ("health mental"[Title/Abstract] OR "mental hygiene"[Title/Abstract] OR "hygiene mental"[Title/Abstract] OR "depressive symptoms"[Title/Abstract] OR "depressive symptom"[Title/Abstract] OR "symptom depressive"[Title/Abstract] OR "emotional depression"[Title/Abstract] OR "depression emotional"[Title/Abstract] OR "Angst"[Title/Abstract] OR "Nervousness"[Title/Abstract] OR "Hypervigilance"[Title/Abstract] OR "Hypervigilance"[Title/Abstract] OR (("Anxiety"[MeSH Terms] OR "Anxiety"[All Fields] OR "Anxieties"[All Fields] OR "anxiety s"[All Fields]) AND "Social"[Title/Abstract]) OR "anxiety social"[Title/Abstract] OR "social anxieties"[Title/Abstract] OR "Anxiousness"[Title/Abstract] OR "Emotion"[Title/Abstract] OR "Feelings"[Title/Abstract] OR "Feeling"[Title/Abstract] OR "Regret"[Title/Abstract] OR "Regrets"[Title/Abstract] OR "Happinesses"[Title/Abstract] OR "Joy"[Title/Abstract] OR "Angers"[Title/Abstract])) | 2,014,915 | 23:17:33 | 2025/9/20 |
| 9 | (((("Mental Health"[Mesh]) OR "Depression"[Mesh]) OR "Anxiety"[Mesh]) OR "Emotions"[Mesh]) OR ((((((((((((((((((((((((Health, Mental[Title/Abstract]) OR (Mental Hygiene[Title/Abstract])) OR (Hygiene, Mental[Title/Abstract])) OR (Depressive Symptoms[Title/Abstract])) OR (Depressive Symptom[Title/Abstract])) OR (Symptom, Depressive[Title/Abstract])) OR (Emotional Depression[Title/Abstract])) OR (Depression, Emotional[Title/Abstract])) OR (Angst[Title/Abstract])) OR (Nervousness[Title/Abstract])) OR (Hypervigilance[Title/Abstract])) OR (Hypervigilance[Title/Abstract])) OR (Anxieties, Social[Title/Abstract])) OR (Anxiety, Social[Title/Abstract])) OR (Social Anxieties[Title/Abstract])) OR (Anxiousness[Title/Abstract])) OR (Emotion[Title/Abstract])) OR (Feelings[Title/Abstract])) OR (Feeling[Title/Abstract])) OR (Regret[Title/Abstract])) OR (Regrets[Title/Abstract])) OR (Happinesses[Title/Abstract])) OR (Joy[Title/Abstract])) OR (Angers[Title/Abstract])) | Publication Date |  | "Mental Health"[MeSH Terms] OR "Depression"[MeSH Terms] OR "Anxiety"[MeSH Terms] OR "Emotions"[MeSH Terms] OR ("health mental"[Title/Abstract] OR "mental hygiene"[Title/Abstract] OR "hygiene mental"[Title/Abstract] OR "depressive symptoms"[Title/Abstract] OR "depressive symptom"[Title/Abstract] OR "symptom depressive"[Title/Abstract] OR "emotional depression"[Title/Abstract] OR "depression emotional"[Title/Abstract] OR "Angst"[Title/Abstract] OR "Nervousness"[Title/Abstract] OR "Hypervigilance"[Title/Abstract] OR "Hypervigilance"[Title/Abstract] OR (("Anxiety"[MeSH Terms] OR "Anxiety"[All Fields] OR "Anxieties"[All Fields] OR "anxiety s"[All Fields]) AND "Social"[Title/Abstract]) OR "anxiety social"[Title/Abstract] OR "social anxieties"[Title/Abstract] OR "Anxiousness"[Title/Abstract] OR "Emotion"[Title/Abstract] OR "Feelings"[Title/Abstract] OR "Feeling"[Title/Abstract] OR "Regret"[Title/Abstract] OR "Regrets"[Title/Abstract] OR "Happinesses"[Title/Abstract] OR "Joy"[Title/Abstract] OR "Angers"[Title/Abstract]) | 701,263 | 23:16:47 | 2025/9/20 |
| 8 | (("Martial Arts"[Mesh]) OR "Tai Ji"[Mesh]) OR ((((((((((((((((((((((Arts, Martial[Title/Abstract]) OR (Judo[Title/Abstract])) OR (Karate[Title/Abstract])) OR (Kung Fu[Title/Abstract])) OR (Gongfu[Title/Abstract])) OR (Gong Fu[Title/Abstract])) OR (Fu, Gong[Title/Abstract])) OR (Tae Kwon Do[Title/Abstract])) OR (Wushu[Title/Abstract])) OR (Hap Ki Do[Title/Abstract])) OR (Aikid[Title/Abstract])) OR (Jujitsu[Title/Abstract])) OR (Tai-ji[Title/Abstract])) OR (Tai Chi[Title/Abstract])) OR (Chi, Tai[Title/Abstract])) OR (Tai Chi Chuan[Title/Abstract])) OR (Taiji[Title/Abstract])) OR (Taijiqun[Title/Abstract])) OR (T'ai Chi[Title/Abstract])) OR (Tai Ji Quan[Title/Abstract])) OR (Ji Quan, Tai[Title/Abstract])) OR (Quan, Tai Ji[Title/Abstract])) | Publication Date |  | "Martial Arts"[MeSH Terms] OR "tai ji"[MeSH Terms] OR ("arts martial"[Title/Abstract] OR "Judo"[Title/Abstract] OR "Karate"[Title/Abstract] OR "kung fu"[Title/Abstract] OR "Gongfu"[Title/Abstract] OR "gong fu"[Title/Abstract] OR "fu gong"[Title/Abstract] OR "tae kwon do"[Title/Abstract] OR "Wushu"[Title/Abstract] OR ("Hap"[All Fields] AND "ki do"[Title/Abstract]) OR "Jujitsu"[Title/Abstract] OR "tai ji"[Title/Abstract] OR "tai chi"[Title/Abstract] OR "chi tai"[Title/Abstract] OR "tai chi chuan"[Title/Abstract] OR "Taiji"[Title/Abstract] OR "t ai chi"[Title/Abstract] OR "tai ji quan"[Title/Abstract] OR "ji quan tai"[Title/Abstract] OR "quan tai ji"[Title/Abstract]) | 6,226 | 23:16:11 | 2025/9/20 |
| 7 | (((((("Executive Function"[Mesh]) OR "Attention"[Mesh]) OR "Memory"[Mesh]) OR "Recognition, Psychology"[Mesh]) OR "Cognition"[Mesh]) OR "Cognitive Flexibility"[Mesh]) OR (((((((((((((((((((((((((((((((((Focus of Attention[Title/Abstract]) OR (Attention Focus[Title/Abstract])) OR (Mental Concentration[Title/Abstract])) OR (Concentration, Mental[Title/Abstract])) OR (Social Attention[Title/Abstract])) OR (Attention, Social[Title/Abstract])) OR (Selective Attention[Title/Abstract])) OR (Attention, Selective[Title/Abstract])) OR (Executive Functions[Title/Abstract])) OR (Function, Executive[Title/Abstract])) OR (Functions, Executive[Title/Abstract])) OR (Executive Control[Title/Abstract])) OR (Executive Controls[Title/Abstract])) OR (Psychology Recognition[Title/Abstract])) OR (Psychological Recognition[Title/Abstract])) OR (Recognition, Psychological[Title/Abstract])) OR (Recognition (Psychology[Title/Abstract]))) OR (Familiarity[Title/Abstract])) OR (Cognitions[Title/Abstract])) OR (Cognitive Function[Title/Abstract])) OR (Cognitive Functions[Title/Abstract])) OR (Function, Cognitive[Title/Abstract])) OR (Functions, Cognitive[Title/Abstract])) OR (Insight[Title/Abstract])) OR (Insights[Title/Abstract])) OR (Cognitive Flexibilities[Title/Abstract])) OR (Cognitive Flexibilities[Title/Abstract])) OR (Flexibility, Cognitive[Title/Abstract])) OR (Flexibility, Cognitive[Title/Abstract])) OR (Cognitive Inflexibilities[Title/Abstract])) OR (Inflexibilities, Cognitive[Title/Abstract])) OR (Inflexibility, Cognitive[Title/Abstract]))) | Publication Date |  | "Executive Function"[MeSH Terms] OR "Attention"[MeSH Terms] OR "Memory"[MeSH Terms] OR "recognition, psychology"[MeSH Terms] OR "Cognition"[MeSH Terms] OR "Cognitive Flexibility"[MeSH Terms] OR ("focus of attention"[Title/Abstract] OR "attention focus"[Title/Abstract] OR "mental concentration"[Title/Abstract] OR "concentration mental"[Title/Abstract] OR "social attention"[Title/Abstract] OR "attention social"[Title/Abstract] OR "selective attention"[Title/Abstract] OR "attention selective"[Title/Abstract] OR "executive functions"[Title/Abstract] OR "function executive"[Title/Abstract] OR "functions executive"[Title/Abstract] OR "executive control"[Title/Abstract] OR "executive controls"[Title/Abstract] OR "psychology recognition"[Title/Abstract] OR "psychological recognition"[Title/Abstract] OR "recognition psychological"[Title/Abstract] OR (("recognition, psychology"[MeSH Terms] OR ("Recognition"[All Fields] AND "Psychology"[All Fields]) OR "psychology recognition"[All Fields] OR "Recognition"[All Fields] OR "recognitions"[All Fields]) AND "Psychology"[Title/Abstract]) OR "Familiarity"[Title/Abstract] OR "Cognitions"[Title/Abstract] OR "cognitive function"[Title/Abstract] OR "cognitive functions"[Title/Abstract] OR "function cognitive"[Title/Abstract] OR "functions cognitive"[Title/Abstract] OR "Insight"[Title/Abstract] OR "Insights"[Title/Abstract] OR "cognitive flexibilities"[Title/Abstract] OR "cognitive flexibilities"[Title/Abstract] OR "flexibility cognitive"[Title/Abstract] OR "flexibility cognitive"[Title/Abstract] OR (("Cognition"[MeSH Terms] OR "Cognition"[All Fields] OR "Cognitions"[All Fields] OR "Cognitive"[All Fields] OR "cognitively"[All Fields] OR "cognitives"[All Fields]) AND "Inflexibilities"[Title/Abstract]) OR ("Inflexibilities"[All Fields] AND "Cognitive"[Title/Abstract]) OR "inflexibility cognitive"[Title/Abstract]) | 1,401,224 | 23:15:34 | 2025/9/20 |
| 6 | (((((((((((((((((((((((Health, Mental[Title/Abstract]) OR (Mental Hygiene[Title/Abstract])) OR (Hygiene, Mental[Title/Abstract])) OR (Depressive Symptoms[Title/Abstract])) OR (Depressive Symptom[Title/Abstract])) OR (Symptom, Depressive[Title/Abstract])) OR (Emotional Depression[Title/Abstract])) OR (Depression, Emotional[Title/Abstract])) OR (Angst[Title/Abstract])) OR (Nervousness[Title/Abstract])) OR (Hypervigilance[Title/Abstract])) OR (Hypervigilance[Title/Abstract])) OR (Anxieties, Social[Title/Abstract])) OR (Anxiety, Social[Title/Abstract])) OR (Social Anxieties[Title/Abstract])) OR (Anxiousness[Title/Abstract])) OR (Emotion[Title/Abstract])) OR (Feelings[Title/Abstract])) OR (Feeling[Title/Abstract])) OR (Regret[Title/Abstract])) OR (Regrets[Title/Abstract])) OR (Happinesses[Title/Abstract])) OR (Joy[Title/Abstract])) OR (Angers[Title/Abstract]) | Publication Date |  | "health mental"[Title/Abstract] OR "mental hygiene"[Title/Abstract] OR "hygiene mental"[Title/Abstract] OR "depressive symptoms"[Title/Abstract] OR "depressive symptom"[Title/Abstract] OR "symptom depressive"[Title/Abstract] OR "emotional depression"[Title/Abstract] OR "depression emotional"[Title/Abstract] OR "Angst"[Title/Abstract] OR "Nervousness"[Title/Abstract] OR "Hypervigilance"[Title/Abstract] OR "Hypervigilance"[Title/Abstract] OR (("Anxiety"[MeSH Terms] OR "Anxiety"[All Fields] OR "Anxieties"[All Fields] OR "anxiety s"[All Fields]) AND "Social"[Title/Abstract]) OR "anxiety social"[Title/Abstract] OR "social anxieties"[Title/Abstract] OR "Anxiousness"[Title/Abstract] OR "Emotion"[Title/Abstract] OR "Feelings"[Title/Abstract] OR "Feeling"[Title/Abstract] OR "Regret"[Title/Abstract] OR "Regrets"[Title/Abstract] OR "Happinesses"[Title/Abstract] OR "Joy"[Title/Abstract] OR "Angers"[Title/Abstract] | 306,306 | 23:14:57 | 2025/9/20 |
| 5 | ((("Mental Health"[Mesh]) OR "Depression"[Mesh]) OR "Anxiety"[Mesh]) OR "Emotions"[Mesh] | Most Recent |  | "Mental Health"[MeSH Terms] OR "Depression"[MeSH Terms] OR "Anxiety"[MeSH Terms] OR "Emotions"[MeSH Terms] | 521,468 | 23:14:24 | 2025/9/20 |
| 4 | (((((((((((((((((((((Arts, Martial[Title/Abstract]) OR (Judo[Title/Abstract])) OR (Karate[Title/Abstract])) OR (Kung Fu[Title/Abstract])) OR (Gongfu[Title/Abstract])) OR (Gong Fu[Title/Abstract])) OR (Fu, Gong[Title/Abstract])) OR (Tae Kwon Do[Title/Abstract])) OR (Wushu[Title/Abstract])) OR (Hap Ki Do[Title/Abstract])) OR (Aikid[Title/Abstract])) OR (Jujitsu[Title/Abstract])) OR (Tai-ji[Title/Abstract])) OR (Tai Chi[Title/Abstract])) OR (Chi, Tai[Title/Abstract])) OR (Tai Chi Chuan[Title/Abstract])) OR (Taiji[Title/Abstract])) OR (Taijiqun[Title/Abstract])) OR (T'ai Chi[Title/Abstract])) OR (Tai Ji Quan[Title/Abstract])) OR (Ji Quan, Tai[Title/Abstract])) OR (Quan, Tai Ji[Title/Abstract]) | Publication Date |  | "arts martial"[Title/Abstract] OR "Judo"[Title/Abstract] OR "Karate"[Title/Abstract] OR "kung fu"[Title/Abstract] OR "Gongfu"[Title/Abstract] OR "gong fu"[Title/Abstract] OR "fu gong"[Title/Abstract] OR "tae kwon do"[Title/Abstract] OR "Wushu"[Title/Abstract] OR ("Hap"[All Fields] AND "ki do"[Title/Abstract]) OR "Jujitsu"[Title/Abstract] OR "Tai-ji"[Title/Abstract] OR "tai chi"[Title/Abstract] OR "chi tai"[Title/Abstract] OR "tai chi chuan"[Title/Abstract] OR "Taiji"[Title/Abstract] OR "t ai chi"[Title/Abstract] OR "tai ji quan"[Title/Abstract] OR "ji quan tai"[Title/Abstract] OR "quan tai ji"[Title/Abstract] | 5,035 | 22:35:59 | 2025/9/20 |
| 3 | ("Martial Arts"[Mesh]) OR "Tai Ji"[Mesh] | Most Recent |  | "Martial Arts"[MeSH Terms] OR "Tai Ji"[MeSH Terms] | 3,742 | 22:35:39 | 2025/9/20 |
| 2 | ((((((((((((((((((((((((((((((((Focus of Attention[Title/Abstract]) OR (Attention Focus[Title/Abstract])) OR (Mental Concentration[Title/Abstract])) OR (Concentration, Mental[Title/Abstract])) OR (Social Attention[Title/Abstract])) OR (Attention, Social[Title/Abstract])) OR (Selective Attention[Title/Abstract])) OR (Attention, Selective[Title/Abstract])) OR (Executive Functions[Title/Abstract])) OR (Function, Executive[Title/Abstract])) OR (Functions, Executive[Title/Abstract])) OR (Executive Control[Title/Abstract])) OR (Executive Controls[Title/Abstract])) OR (Psychology Recognition[Title/Abstract])) OR (Psychological Recognition[Title/Abstract])) OR (Recognition, Psychological[Title/Abstract])) OR (Recognition (Psychology[Title/Abstract]))) OR (Familiarity[Title/Abstract])) OR (Cognitions[Title/Abstract])) OR (Cognitive Function[Title/Abstract])) OR (Cognitive Functions[Title/Abstract])) OR (Function, Cognitive[Title/Abstract])) OR (Functions, Cognitive[Title/Abstract])) OR (Insight[Title/Abstract])) OR (Insights[Title/Abstract])) OR (Cognitive Flexibilities[Title/Abstract])) OR (Cognitive Flexibilities[Title/Abstract])) OR (Flexibility, Cognitive[Title/Abstract])) OR (Flexibility, Cognitive[Title/Abstract])) OR (Cognitive Inflexibilities[Title/Abstract])) OR (Inflexibilities, Cognitive[Title/Abstract])) OR (Inflexibility, Cognitive[Title/Abstract])) | Publication Date |  | "focus of attention"[Title/Abstract] OR "attention focus"[Title/Abstract] OR "mental concentration"[Title/Abstract] OR "concentration mental"[Title/Abstract] OR "social attention"[Title/Abstract] OR "attention social"[Title/Abstract] OR "selective attention"[Title/Abstract] OR "attention selective"[Title/Abstract] OR "executive functions"[Title/Abstract] OR "function executive"[Title/Abstract] OR "functions executive"[Title/Abstract] OR "executive control"[Title/Abstract] OR "executive controls"[Title/Abstract] OR "psychology recognition"[Title/Abstract] OR "psychological recognition"[Title/Abstract] OR "recognition psychological"[Title/Abstract] OR (("recognition, psychology"[MeSH Terms] OR ("Recognition"[All Fields] AND "Psychology"[All Fields]) OR "psychology recognition"[All Fields] OR "Recognition"[All Fields] OR "recognitions"[All Fields]) AND "Psychology"[Title/Abstract]) OR "Familiarity"[Title/Abstract] OR "Cognitions"[Title/Abstract] OR "cognitive function"[Title/Abstract] OR "cognitive functions"[Title/Abstract] OR "function cognitive"[Title/Abstract] OR "functions cognitive"[Title/Abstract] OR "Insight"[Title/Abstract] OR "Insights"[Title/Abstract] OR "cognitive flexibilities"[Title/Abstract] OR "cognitive flexibilities"[Title/Abstract] OR "flexibility cognitive"[Title/Abstract] OR "flexibility cognitive"[Title/Abstract] OR (("cognition"[MeSH Terms] OR "cognition"[All Fields] OR "Cognitions"[All Fields] OR "Cognitive"[All Fields] OR "cognitively"[All Fields] OR "cognitives"[All Fields]) AND "Inflexibilities"[Title/Abstract]) OR ("Inflexibilities"[All Fields] AND "Cognitive"[Title/Abstract]) OR "inflexibility cognitive"[Title/Abstract] | 1,044,991 | 22:34:56 | 2025/9/20 |
| 1 | ((((("Executive Function"[Mesh]) OR "Attention"[Mesh]) OR "Memory"[Mesh]) OR "Recognition, Psychology"[Mesh]) OR "Cognition"[Mesh]) OR "Cognitive Flexibility"[Mesh] | Most Recent |  | "Executive Function"[MeSH Terms] OR "Attention"[MeSH Terms] OR "Memory"[MeSH Terms] OR "recognition, psychology"[MeSH Terms] OR "Cognition"[MeSH Terms] OR "Cognitive Flexibility"[MeSH Terms] | 425,738 | 22:34:27 | 2025/9/20 |

## Embase（4325）

| **No.** | **Query** | **Results** |
| --- | --- | --- |
| #11 | #7 AND #10 | 4325 |
| #10 | #8 OR #9 | 5494081 |
| #9 | #5 OR #6 | 2118024 |
| #8 | #3 OR #4 | 4101240 |
| #7 | #1 OR #2 | 9545 |
| #6 | 'condition, mental':ab,ti OR 'health, mental':ab,ti OR 'mental care':ab,ti OR 'mental condition':ab,ti OR 'mental factor':ab,ti OR 'mental help':ab,ti OR 'mental service':ab,ti OR 'mental state':ab,ti OR 'mental status':ab,ti OR 'mental status schedule':ab,ti OR 'psychic health':ab,ti OR 'mental health':ab,ti OR 'central depression':ab,ti OR 'clinical depression':ab,ti OR 'depressive disease':ab,ti OR 'depressive disorder':ab,ti OR 'depressive episode':ab,ti OR 'depressive illness':ab,ti OR 'depressive personality disorder':ab,ti OR 'depressive state':ab,ti OR 'depressive symptom':ab,ti OR 'depressive syndrome':ab,ti OR 'depressivity':ab,ti OR 'mental depression':ab,ti OR 'parental depression':ab,ti OR 'depression':ab,ti OR 'anxiety':ab,ti OR 'emotional factor':ab,ti OR 'emotional response':ab,ti OR 'emotional status':ab,ti OR 'emotional structure':ab,ti OR 'emotions':ab,ti OR 'expressed emotion':ab,ti OR 'emotion':ab,ti | 1329776 |
| #5 | 'mental health'/exp OR 'depression'/exp OR 'anxiety'/exp OR 'emotion'/exp | 1710175 |
| #4 | ('cognitive control':ab,ti OR 'executive control':ab,ti OR 'executive function':ab,ti OR 'attentiveness':ab,ti OR 'attention':ab,ti OR 'item recall':ab,ti OR 'memory function':ab,ti OR 'nonspatial memory':ab,ti OR 'remembering':ab,ti OR 'reminiscence':ab,ti OR 'memory':ab,ti OR 'form recognition':ab,ti OR recognition:ab,ti) AND psychology:ab,ti OR 'recognition, psychology':ab,ti OR 'recognition':ab,ti OR 'cognitive accessibility':ab,ti OR 'cognitive balance':ab,ti OR 'cognitive dissonance':ab,ti OR 'cognitive function':ab,ti OR 'cognitive functioning':ab,ti OR 'cognitive structure':ab,ti OR 'cognitive symptoms':ab,ti OR 'cognitive task':ab,ti OR 'cognitive thinking':ab,ti OR 'neurobehavioural manifestations':ab,ti OR 'volition':ab,ti OR 'cognition':ab,ti OR 'cognitive flexibility':ab,ti | 793309 |
| #3 | 'executive function'/exp OR 'attention'/exp OR 'memory'/exp OR 'recognition'/exp OR 'cognition'/exp OR 'cognitive flexibility'/exp | 3645909 |
| #2 | ((('martial arts':ab,ti OR 'martial sport':ab,ti OR 'martial art':ab,ti OR 'aikido':ab,ti OR 'ju jitsu':ab,ti OR 'ju jutsu':ab,ti OR 'jujutsu':ab,ti OR 'jiu jitsu':ab,ti OR 'judo':ab,ti OR 'karateka':ab,ti OR 'karatekas':ab,ti OR 'karate':ab,ti OR 'kungfu':ab,ti OR 'kung fu':ab,ti OR 'chi kung':ab,ti OR 'chigung':ab,ti OR 'qi gong':ab,ti OR 'qigong':ab,ti OR 'tae kwon do':ab,ti OR 'taekwondo':ab,ti OR 'tai chi chuan':ab,ti OR 'tai ji':ab,ti OR 'taiji quan':ab,ti OR 'taijiquan':ab,ti OR 'tai chi':ab,ti OR sanda:ab,ti) AND sport:ab,ti OR taolu:ab,ti) AND sport:ab,ti OR wushu:ab,ti) AND sport:ab,ti | 1019 |
| #1 | 'martial art'/exp OR 'aikido'/exp OR 'jiu jitsu'/exp OR 'judo'/exp OR 'karate'/exp OR 'kung fu'/exp OR 'qigong'/exp OR 'taekwondo'/exp OR 'tai chi'/exp OR 'wushu (sport)'/exp | 9213 |

## **Cochrane Library（991）**

ID Search Hits

#1 MeSH descriptor: [Martial Arts] explode all trees 840

#2 (Wushu or Jujitsu or Tae Kwon Do or Karate or Aikido or Gongfu or Gong Fu or Fu, Gong or Kung Fu or Arts, Martial or Hap Ki Do or Judo):ti,ab,kw (Word variations have been searched) 662

#3 MeSH descriptor: [Tai Ji] explode all trees 608

#4 (Taijiquan or Tai Chi Chuan or T'ai Chi or Taiji or Tai Chi or Ji Quan, Tai or Quan, Tai Ji or Chi, Tai or Tai-ji or Tai Ji Quan):ti,ab,kw (Word variations have been searched) 2238

#5 MeSH descriptor: [Qigong] explode all trees 182

#6 (Ch'i Kung or Qi Gong):ti,ab,kw (Word variations have been searched) 89

#7 #1 or #2 or #3 or #4 or #5 or #6 with Publication Year from 1950 to 2025, with Cochrane Library publication date Between Jan 1950 and Jul 2025, in Trials 2987

#8 MeSH descriptor: [Executive Function] explode all trees 1962

#9 MeSH descriptor: [Memory, Short-Term] explode all trees 2419

#10 MeSH descriptor: [Inhibition, Psychological] explode all trees 874

#11 MeSH descriptor: [Cognition] explode all trees 16701

#12 MeSH descriptor: [Cognitive Flexibility] explode all trees 3

#13 MeSH descriptor: [Attention] explode all trees 7084

#14 MeSH descriptor: [Memory] explode all trees 10055

#15 (Executive Control or Function, Executive or Executive Functions or Functions, Executive or Executive Controls or Insight or Insights or Cognitions or Function, Cognitive or Functions, Cognitive or Cognitive Function or Cognitive Functions or Psychological Inhibitions or Psychology Inhibition or Inhibitions (Psychology) or Psychology Inhibitions or Inhibitions, Psychology or Inhibition, Psychology or Psychological Inhibition or Inhibitions, Psychological or Inhibition (Psychology) or Memories, Shortterm or Short-Term Memory or Working Memory or Immediate Memories or Working Memories or Shortterm Memories or Short-Term Memories or Memory, Shortterm or Memory, Short Term or Immediate Memory or Shortterm Memory or Memory, Immediate or Memories, Short-Term or Memories, Immediate or Recall, Immediate or Recalls, Immediate or Immediate Recalls or Immediate Recall or Flexibilities, Cognitive or Flexibility, Cognitive or Cognitive Flexibilities or Cognitive Inflexibilities or Inflexibilities, Cognitive or Cognitive Inflexibility or Inflexibility, Cognitive):ti,ab,kw (Word variations have been searched) 151214

#16 #8 or #9 or #10 or #11 or #12 or #13 or #14 or #15 160454

#17 MeSH descriptor: [Mental Health] explode all trees 3548

#18 MeSH descriptor: [Depression] explode all trees 19523

#19 MeSH descriptor: [Anxiety] explode all trees 13616

#20 (Mental Hygiene OR Hygiene, Mental OR Health, Mental OR Emotional Depression OR Depressive Symptoms OR Depressive Symptom OR Symptom, Depressive OR Depression, Emotional OR Angst OR Social Anxieties OR Anxiety, Social OR Social Anxiety OR Anxieties, Social OR Nervousness OR Anxiousness OR Hypervigilance):ti,ab,kw 85614

#21 #17 or #18 or #19 or #20 101916

#22 #16 or #21 231273

#23 #7 and #22 991

## **Web of Science Core Collection（1050）**

TS=("executive function*" OR "executive control*" OR attention OR "selective attention" OR "social attention" OR "focus of attention" OR "attention focus" OR "mental concentration" OR "concentration mental" OR memory OR cognition OR cognitions OR "cognitive function*" OR insight OR insights OR "cognitive flexibilit*" OR "cognitive inflexibilit*" OR "recognition psychology" OR "psychological recognition" OR familiarity OR "mental health" OR "depressive symptom*") AND TS=("martial art*" OR judo OR karate OR "kung fu" OR gongfu OR "gong fu" OR "tae kwon do" OR taekwondo OR wushu OR "hap ki do" OR jujitsu OR "tai ji" OR taiji OR "tai chi" OR "tai chi chuan" OR taijiquan OR "t'ai chi" OR "tai ji quan")

## **Ovid MEDLINE（876）**

| Ovid MEDLINE(R) ALL <1946 to September 20, 2025> |  |  |
| --- | --- | --- |
| 1 | Executive Function.mp. or exp *Executive Function/ | 38952 |
| 2 | Attention.mp. or exp *Attention/ | 719541 |
| 3 | Memory.mp. or exp *Memory/ | 407676 |
| 4 | Recognition, Psychology.mp. or exp *Recognition, Psychology/ | 23193 |
| 5 | Cognition.mp. or exp *Cognition/ | 325733 |
| 6 | Cognitive Flexibility.mp. or exp *Cognitive Flexibility/ | 6073 |
| 7 | (executive function or executive functions or function, executive or functions, executive or executive control or executive controls or attention or focus ofattention focus or mental concentration or concentration, mental or socialattention, social or selectiveattention, selective or memory or cognition or cognitions or cognitive function or cognitive functions or function, cognitive or functions, cognitive or insight or insights or cognitive flexibility or cognitive flexibilities or flexibilities, cognitive or flexibility, cognitive or cognitive inflexibility or cognitive inflexibilities or inflexibilities, cognitive or inflexibility, cognitive).ab,ti,kw. | 1979865 |
| 8 | martial arts.mp. or exp *Martial Arts/ | 4270 |
| 9 | taiji.mp. or exp *Tai Ji/ | 1584 |
| 10 | (martial arts or arts, martial or judo or karate or kung fu or gongfu or gong fu or fu, gong or tae kwon do or wushu or hap ki do or aikido or jujitsu or tai ji or tai-ji or tai chi or chi, tai or tai chi chuan or taiji or taijiquan or t'ai chi or tai ji quan or ji quan, tai or quan, tai ji).ab,ti,kw. | 6063 |
| 11 | 1 or 2 or 3 or 4 or 5 or 6 or 7 | 2186855 |
| 12 | 8 or 9 or 10 | 6789 |
| 13 | 11 and 12 | 876 |

## **CNKI（850）**

AB = (taijiquan + taiji + taiji exercise + taiji exercise + taiji exercise + taiji teaching + taiji push hands + wushu + wushu exercise + wushu sanshou + chinese wushu + traditional wushu + judo + judo exercise + judo training + judo teaching + hapkido + karate + karate exercise + karate training + tae kwon do + tae kwon do training + tae kwon do teaching + wrestling + wrestling exercise + chinese style wrestling + wrestling training + freestyle Wrestling + Kung Fu + Chinese Kung Fu + Qigong + Qigong Therapy + Qigong Ba Duan Jin + Ba Duan Jin Training + Wu An Qiu Opera + Yi Jin Jing) AND SU = ("executive function*" + "executive control*" OR attention + "selective attention" + "social attention" + "focus of attention" + "attention focus" + "mental concentration" + "concentration mental" + memory + cognition + cognitions + "cognitive function*" + insight + insights + "cognitive flexibilit*" + "cognitive inflexibilit*" + "recognition psychology" + "psychological recognition" + familiarity + "mental health" + "depressive symptom*" + "emotional depression" + depression)

# Supplementary S3: Funnel plot of the effect sizes for depressive symptoms


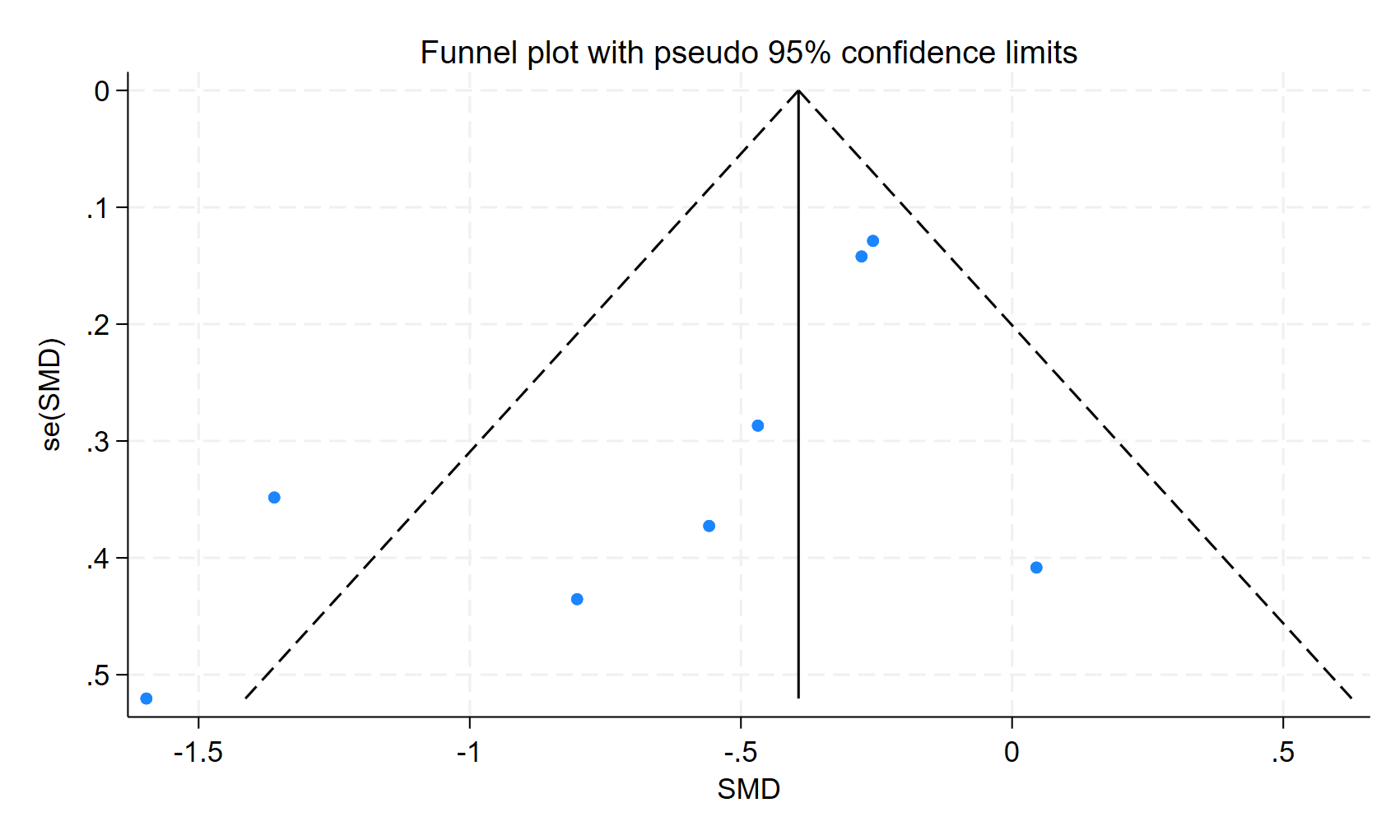


# Supplementary S4: Funnel plot of the effect sizes for Cognitive Function


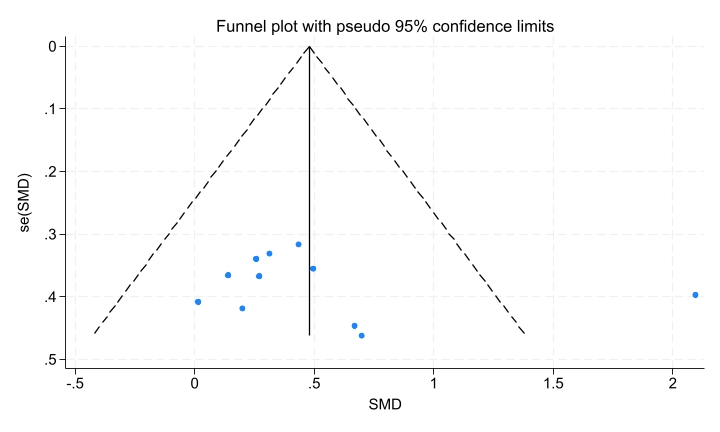


# Supplementary S5: Sensitivity analysis for depressive symptoms.


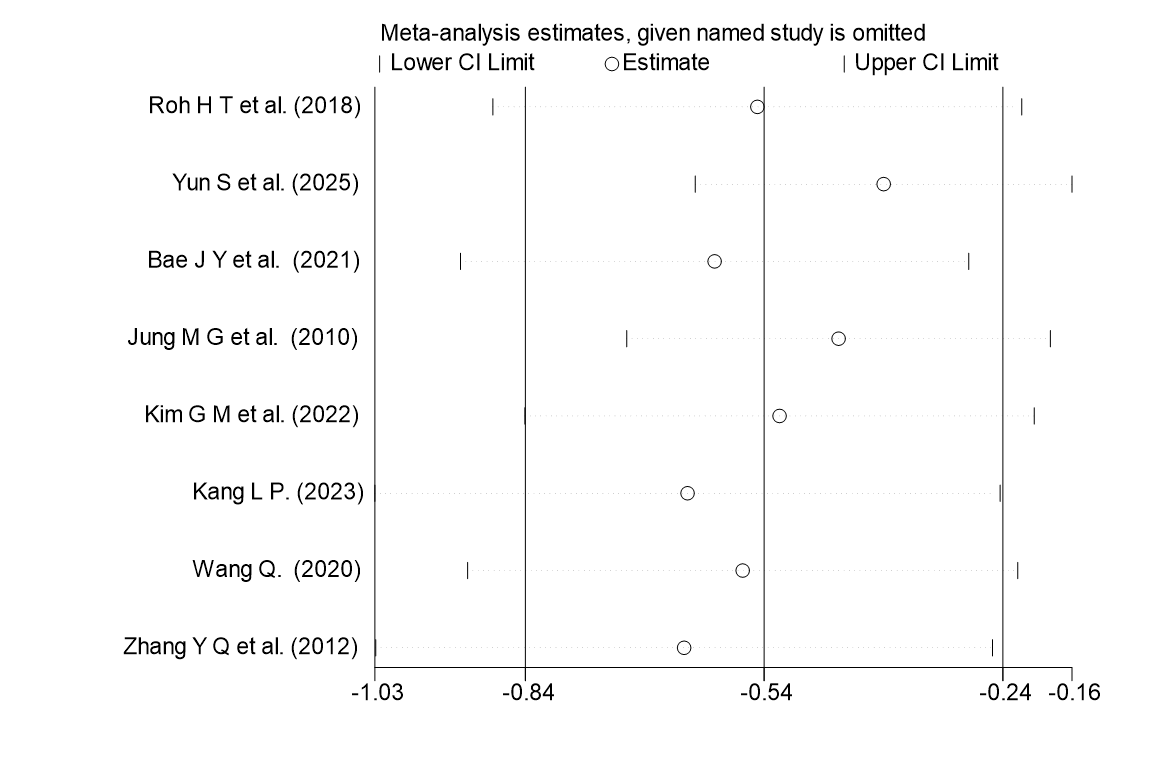


# Supplementary S6: Sensitivity analysis for cognitive function.


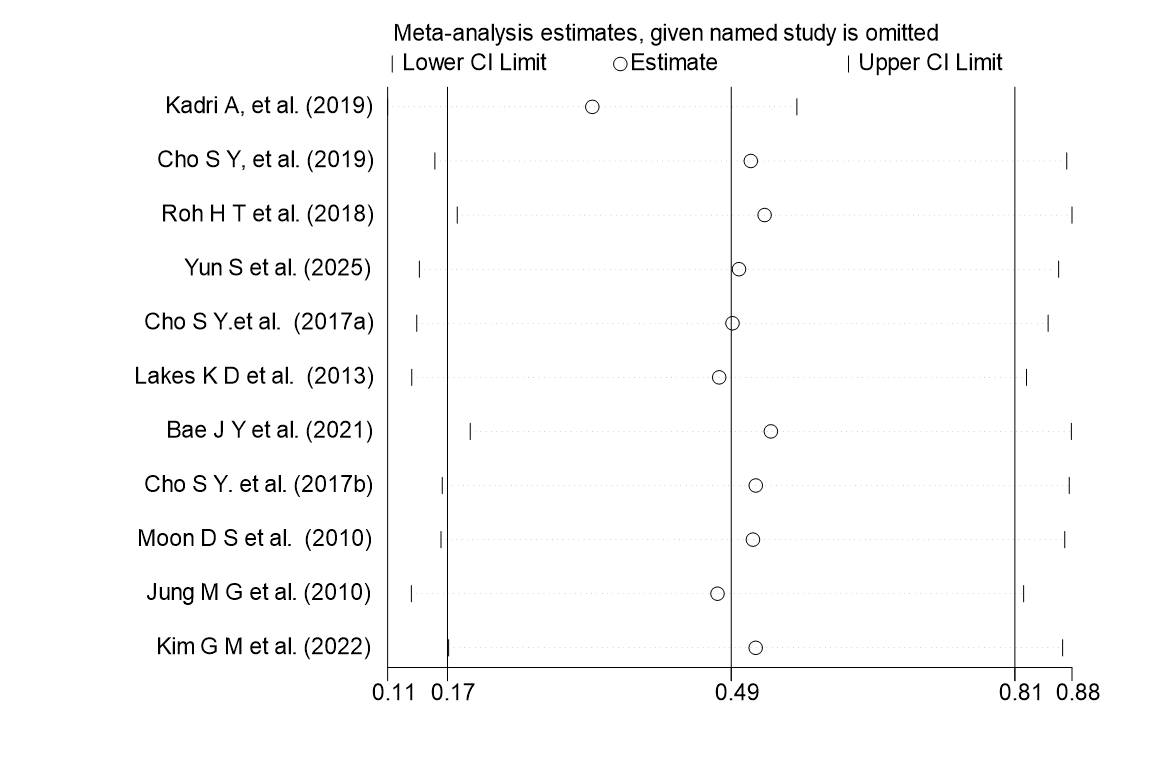


# Supplementary S7: Forest plot of cognitive function after excluding outlier studies

**
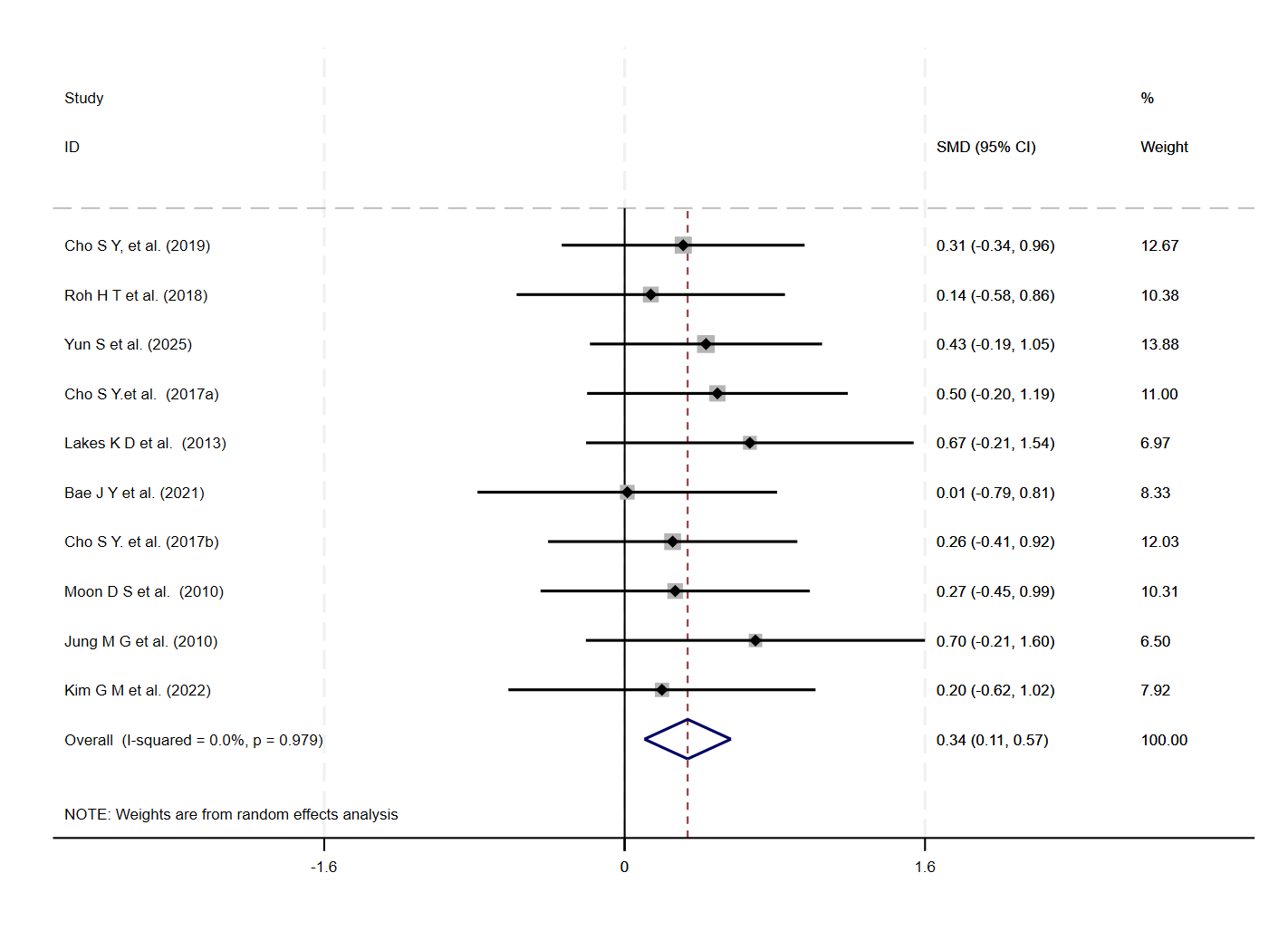
**

**Supplementary S8: GRADE Quality Assessment Table**

| **Certainty assessment** | | | | | | | **№ of patients** | | **Effect** | | **Certainty** | **Importance** |
| --- | --- | --- | --- | --- | --- | --- | --- | --- | --- | --- | --- | --- |
| **№ of studies** | **Study design** | **Risk of bias** | **Inconsistency** | **Indirectness** | **Imprecision** | **Other considerations** | **Taekwondo** | **No-Taekwondo** | **Relative (95% CI)** | **Absolute (95% CI)** |  |  |
| **Depression Symptoms (assessed with: Self-Rating Depression Scale; Middle School Students' Mental Health Scale; Korean Profile of Mood States - Brief; Beck Depression Inventory-II; Korean Mini-Mental State Examination)** | | | | | | | | | | | | |
| 643 | randomised trials | serious^a^ | not serious | serious^b^ | not serious | strong association | 348 | 295 | - | **0**  (0.84 lower to 0.24 lower) | ⨁⨁⨁◯ Moderate^a,b^ |  |
| **Cognitive Function (assessed with: Stroop Color-Word Test;Hearts & Flowers Test)** | | | | | | | | | | | | |
| 335 | randomised trials | serious^c^ | not serious | serious^d^ | not serious | strong association | 166 | 169 | - | **0**  (0.17 higher to 0.81 higher) | ⨁⨁⨁◯ Moderate^c,d^ |  |

**CI:** confidence interval

**Explanations**

a. Most studies demonstrated high risk of bias in more than one area of study methodologya.

b. population heterogeneity affecting applicability.

c. Most studies demonstrated high risk of bias in more than one area of study methodologya.

d. population heterogeneity affecting applicability
